# Massage‐Mimicking Nanosheets Mechanically Reorganize Inter‐organelle Contacts to Restore Mitochondrial Functions in Parkinson's Disease

**DOI:** 10.1002/advs.202413376

**Published:** 2025-04-13

**Authors:** Tianqi Li, Liwen Huang, Chenxiao Guo, Jing Ren, Xi Chen, Yachu Ke, Zengyu Xun, Wenzhuo Hu, Yilin Qi, Heping Wang, Zhongying Gong, Xing‐Jie Liang, Xue Xue

**Affiliations:** ^1^ State Key Laboratory of Medicinal Chemical Biology College of Pharmacy Nankai University Tianjin 300350 P. R. China; ^2^ Department of Neurology Tianjin First Central Hospital School of Medicine Nankai University Tianjin 300192 P. R. China; ^3^ Laboratory of Controllable Nanopharmaceuticals Chinese Academy of Sciences (CAS) Center for Excellence in Nanoscience and CAS Key Laboratory for Biomedical Effects of Nanomaterials and Nanosafety National Center for Nanoscience and Technology Beijing 100190 P. R. China; ^4^ University of Chinese Academy of Sciences Beijing 101408 P. R. China; ^5^ Present address: Department of Chemistry Shanghai Key Laboratory of Molecular Catalysis and Innovative Materials State Key Laboratory of Molecular Engineering of Polymers and iChem Fudan University Shanghai 200438 P. R. China; ^6^ Present address: State Key Laboratory of Advanced Medical Materials and Devices Tianjin Key Laboratory of Radiation Medicine and Molecular Nuclear Medicine Key Laboratory of Radiopharmacokinetics for Innovative Drugs Tianjin Institutes of Health Science Institute of Radiation Medicine Chinese Academy of Medical Sciences & Peking Union Medical College Tianjin 300192 P. R. China

**Keywords:** inter‐organelle contacts, mechanical stimulation, mitochondria, nanosheets, Parkinson's disease

## Abstract

Parkinson's disease (PD) is exacerbated by dysfunction of inter‐organelle contact, which depends on cellular responses to the mechanical microenvironment and can be regulated by external mechanical forces. Delivering dynamic mechanical forces to neural cells proves challenging due to the skull. Inspired by the effects of massage; here PEGylated black phosphorus nanosheets (PEG‐BPNS), known for their excellent biocompatibility, biodegradability, specific surface area, mechanical strength, and flexibility, are introduced, which are capable of adhering to neural cell membrane and generating mechanical stimulation with their lateral size of 200 nm, exhibiting therapeutic potential in a 1‐methyl‐4‐phenyl‐1,2,3,6‐te‐trahydropyridine‐induced PD mouse model by regulating inter‐organelle contacts. Specifically, it is found that 200 nm PEG‐BPNS, acting as “NanoMassage,” significantly increase  plasma membrane tension, as evidenced by fluorescent lipid tension reporter fluorescence lifetime analysis. This mechanical force modulates actin reorganization, subsequently regulating the contacts between actin, mitochondria, and endoplasmic reticulum, further controlling mitochondrial fission and mitigating mitochondrial dysfunction in PD, exhibiting therapeutic efficacy via intranasal administration. These findings provide a noninvasive strategy for applying mechanical stimulation to deep brain areas and elucidate the mechanism of NanoMassage mediating inter‐organelle contacts, suggesting the rational design of “NanoMassage” to remodel inter‐organelle communications in neurodegenerative disease treatment.

## Graphical abstract

Parkinson's disease (PD) progression is aggravated by disrupted inter‐organelle contacts, which are influenced by mechanical forces. NanoMassage, 200 nm PEGylated black phosphorus nanosheets, deliver targeted mechanical stimulation to neural cells. By enhancing membrane tension and regulating inter‐organelle communication, NanoMassage mitigate mitochondrial dysfunction in PD mice. This approach offers a noninvasive therapeutic strategy for diseases associated with impaired inter‐organelle interactions.

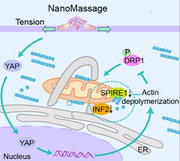

## 1 Introduction

Parkinson's disease (PD) is a neurodegenerative disorder characterized by advanced‐stage motor disabilities and the selective loss of dopaminergic neurons in the substantia nigra (SN) pars compacta.^[^ ^1^ ^]^ The pathogenesis of PD is associated with dysfunction in several cellular processes, including mitochondrial dysfunction, oxidative stress, and impaired protein homeostasis.^[^ ^2^ ^]^ While these factors play a central role in dopaminergic neuron degeneration, emerging evidence suggests that the disruption of inter‐organelle contacts, particularly between mitochondria and the endoplasmic reticulum (ER), may exacerbate these pathological processes by impairing mitochondrial dynamics and function.^[^ ^3^ ^]^ Modulating mitochondria–ER contact sites (MERCs) in dopaminergic neurons represents a promising therapeutic strategy for PD, as it may improve mitochondrial dynamics, restore mitochondrial function, and mitigate oxidative stress.^[^ ^4^ ^]^ Additionally, cellular interactions with the surrounding microenvironment play a critical role in influencing the subcellular distribution and coordination of organelles, thereby affecting their functionality.^[^ ^5^ ^]^ Therefore, a mechano‐mediated approach to locally modulate inter‐organelle contacts in deep brain regions may offer a viable strategy for the treatment of PD.

Current methodologies for delivering mechanical stimulation to cells generally employ two principal approaches: 1) modulating the stiffness or topography of the extracellular matrix (ECM) to indirectly generate mechanical forces, and 2) utilizing specialized devices, such as atomic force microscopy (AFM), optical tweezers, and magnetomechanical‐based techniques, to directly apply localized forces.^[^ ^6^ ^]^ Various in vitro models have been established to generate different types of mechanical forces in cell culture, including biomaterial‐based substrates with distinct mechanical properties,^[^ ^7^ ^]^ fluid flow models that create shear stress, and compression applications using metal coins or lead pellets.^[^ ^8^ ^]^ While able to deliver local forces, these approaches necessitate tissue–surface contact and are limited to external force stimulation.^[^ ^9^ ^]^ In 3D developing tissues, force generation is not confined to the surface, necessitating the capability to provide internal mechanical stimulation. The intricacies of ECM composition and the specific demands of these devices hinder their effective transition from in vitro systems to in vivo contexts. To address these limitations, noninvasive strategies for applying mechanical forces have been developed. Magnetic materials that respond to magnetic fields and convert these signals into mechanical forces have emerged as a promising solution for noninvasively and precisely controlling the forces exerted on cells. For example, specific biomolecule‐tethered magnetic material can generate mechanical forces under the control of a magnetic field setup to the associated or attached cells.^[^ ^10^ ^]^ Similarly, cell‐laden scaffolds responsive to magnetic fields have been employed to regulate granulation tissue contractility and myofibroblast differentiation.^[^ ^11^ ^]^ Although magnetic fields are considered promising patient‐friendly therapeutic strategies for various diseases, intracellular components such as magnetic protein biocompasses and ferritin containing iron atoms can be sensitive to these fields. This sensitivity may inadvertently exert excessive forces on normal tissues, resulting in divergent cellular responses that limit their clinical applicability.^[^ ^12^ ^]^ Consequently, there is a pressing need for noninvasive strategies to deliver mechanical forces to neural cells without adversely affecting surrounding healthy tissues,^[^ ^13^ ^]^ enabling targeted mechanical stimulation of deep brain regions to facilitate the treatment of neurological disorders.

Massage therapy is a noninvasive and readily accessible intervention that effectively mitigates symptoms of a range of diseases such as musculoskeletal injuries, chronic pain, anxiety, and certain neurological disorders.^[^ ^14^ ^]^ By applying mechanical forces to the plasma membrane (PM) of cells, massage therapy triggers a timely and sustained increase in mechanically sensitive signals within the cellular environment. For instance, by stretching and pulling muscles, massage therapy promotes mitochondrial biogenesis and reduces cellular inflammatory responses, ultimately ameliorating pain and expediting muscle healing.^[^ ^15^ ^]^ Additionally, this process coordinates various essential cellular processes by initially regulating the polymerization and depolymerization of the actin cytoskeleton, and subsequently modulating mitochondrial dynamics and metabolism, inhibiting neural cell apoptosis and ameliorating the symptoms of neurological diseases, etc.^[^ ^16^ ^]^ Despite there are many successful cases of massage therapy in treating numerous conditions, a remarkable challenge persists in producing massage‐mimicking mechanical stimulation to deep brain areas.

Inspired by the beneficial effects of massage, here we aim to introduce “NanoMassage,” a kind of well‐designed nanosheet capable of adhering to the neural cell membrane to produce direct and dynamic interactions with the neural cell membrane, conducting their mechanical properties to affect intracellular neural signals effectively. To efficiently deliver mechanical stimulations to cells, optimizing the adhesion of nanomaterials to cell membranes is pivotal. Nanosheets are uniquely suited for this purpose, offering adjustable adhesion properties that enhance the application of mechanical forces unattainable by other materials. Specifically, the strong in‐plane covalent bonds and atomic thickness of nanosheets confer exceptional mechanical strength and flexibility for interacting with cell membranes. Their large lateral size provides an ultrahigh contact area, while the high exposure of surface atoms facilitates easy regulation of adhesion properties through surface modification and functionalization.^[^ ^17^ ^]^ Recent studies, as well as our previous study,^[^ ^18^ ^]^ have pointed out that nanosheets exhibit remarkable enhancements and unprecedented control over cellular interactions. The sizes of nanosheets significantly affect their interactions with the PM of cells, cellular uptake efficiency, and mode of cell internalization,^[^ ^19^ ^]^ subsequently impacting cellular behaviors.^[^ ^20^ ^]^ Nanosheets with larger diameters demonstrate stronger adherence to the surface of cells, further inducing cytoskeletal reorganization, while smaller diameters are predominantly internalized by cells.^[^ ^18b^ ^]^ Among various kinds of nanosheets, black phosphorus nanosheets (BPNS) are notable for their exceptional biodegradability and biocompatibility, undergoing degradation into nontoxic intermediates, such as phosphate and other phosphorus oxides in vivo.^[^ ^21^ ^]^ Consequently, BPNS have aroused great interest from enormous research fields, including cancer therapy, drug delivery, bioimaging, and biosensing.^[^ ^22^ ^]^ Therefore, we hypothesize that by optimizing the biodegradable BPNS with precise surface chemical engineering, the “NanoMassage” can dynamically interact with cell membrane, thus reorganizing inter‐organelle contacts and potentially alleviating PD pathology.

**Scheme 1:**
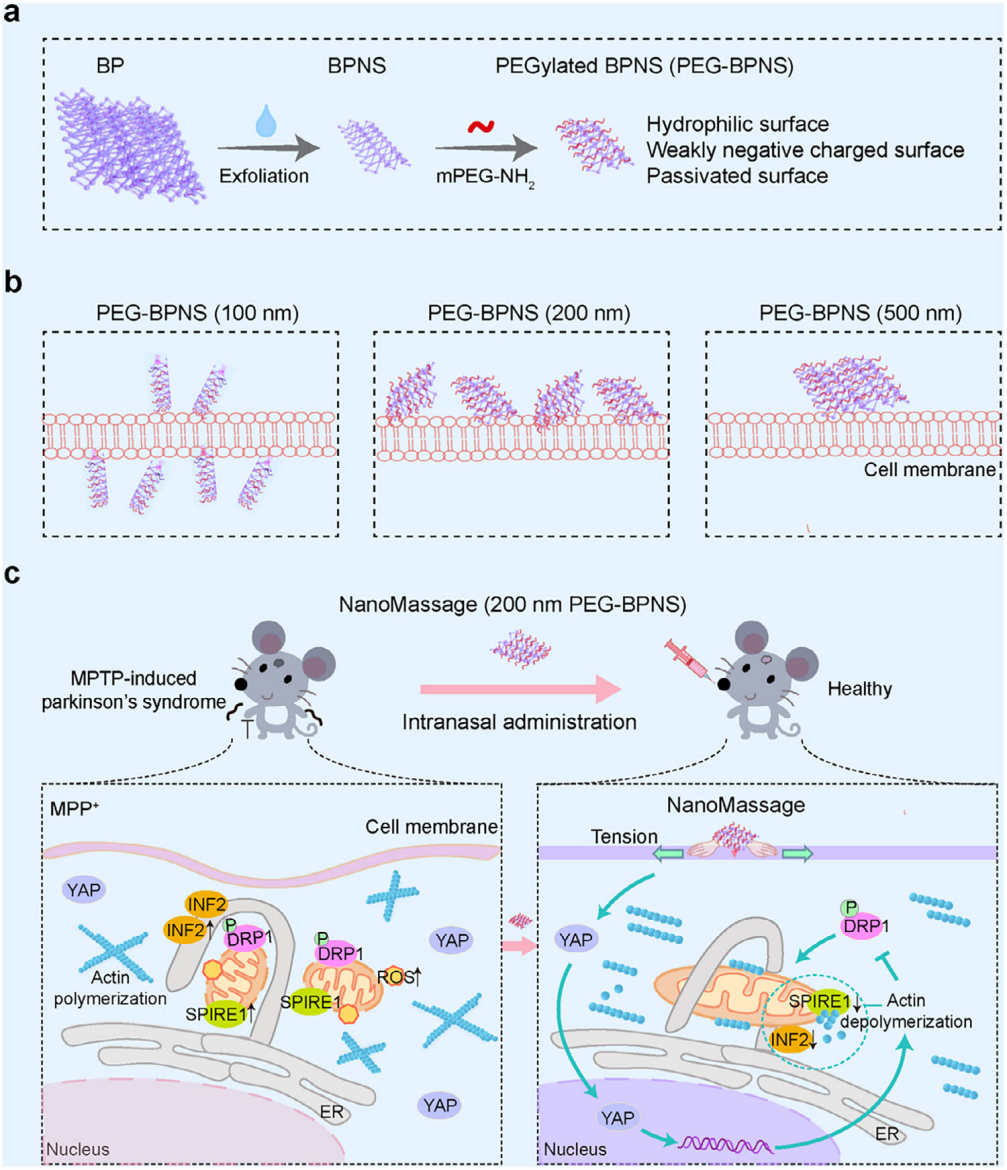
Schematic illustration of the study approach. a) Schematic illustration for the preparation of PEG‐BPNS. b) Schematic illustration for the interaction between PEG‐BPNS of different dimensions and cell membranes. c) Schematic illustration for the mechanism of NanoMassage on mitigating PD symptoms of mice. Following intranasal administration, NanoMassage exhibit accumulated levels in the brains of PD mice, enabling their adherence to the PM of neural cells. Then, NanoMassage induce an elevation in PM tension. This heightened tension in the PM of cells facilitates the translocation of yes‐associated protein (YAP) and subsequently promotes YAP activation. This process facilitates actin filaments depolymerization, disrupting the interaction between Spire1C and inverted formin (INF2) and consequently reducing the levels of these proteins. The resultant restructuring of the cellular actin cytoskeleton hampers the recruitment and assembly of phosphorylated DRP1, thereby fine‐tuning mitochondrial fission. Consequently, the maintenance of mitochondrial morphology, prevention of reduction in mitochondrial membrane potential, and mitigation of reactive oxidative stress (ROS) generation are achieved. Ultimately, the therapeutic effects of NanoMassage manifest in the alleviation of PD symptoms in mice by safeguarding mitochondrial functions in neural cells.

The interaction between nanosheets and cell membranes is affected by two primary factors: 1) the physiochemical properties of nanomaterials, which are determined by their constitutive components, hydrogen bond donors, and surface modifications; 2) the disruption caused by the protein corona in biological environments. To enhance the interaction between nanosheets and cell membranes, we employed polyethylene glycol (PEG) to modify BPNS. PEG forms hydrogen bonds with hydronium ions (H_3_O^+^) in water,^[^ ^23^ ^]^ transforming into a supra‐polyelectrolyte that neutralizes the negative charge of BPNS, thereby enhancing the interaction between PEGylated BPNS (PEG‐BPNS) and cell membranes. Additionally, PEG creates passivated surfaces on PEGylated nanosheets, preventing the absorption of proteins or enzymes, and thereby avoiding cellular internalization due to the formation of a protein corona in vivo. Upon PEG functionalization, “NanoMassage” can be endowed with reduced internalization, prolonged circulation time, and improved in vivo biocompatibility. Therefore, in this study, we prepared biodegradable PEG‐BPNS each with identical surface chemistry but different lateral sizes, including 100, 200, and 500 nm. With a series of well‐designed PEG‐BPNS, we explored their impact on mechanical stimulation, oxidative stress regulation, and cell survival in neural cells, choosing the identical PEG‐BPNS of 200 nm (noted by “NanoMassage”), which induced the increase of PM tension, subsequently reducing oxidative stress and inhibiting the death of neural cells, to treat 1‐methyl‐4‐phenyl‐1,2,3,6‐te‐trahydropyridine (MPTP)‐induced PD mice. Subsequently, the therapeutic effects of NanoMassage on the motor function and various neuropathologies in MPTP‐induced PD mice were evaluated. To clarify the mechanism of NanoMassage in neural cells, we elucidated the mechanistic process, exhibited the inter‐organelle reorganized process, and investigated their downstream pathways. Furthermore, considering the clinical application of NanoMassage in deep brain regions with noninvasive administration, we assessed the effectiveness of intranasal administration of NanoMassage in PD mice (**Scheme** **1**). Our results indicated that modulating inter‐organelle contacts is a mechanism by which NanoMassage alleviate PD symptoms, highlighting the dimension of nanosheet as a tunable physicochemical parameter that could be further leveraged for rational exploitation of mechanical stimulation in future research and therapeutic development.

## 2 Results

### 2.1 Preparation and Identification of NanoMassage

**Figure 1:**
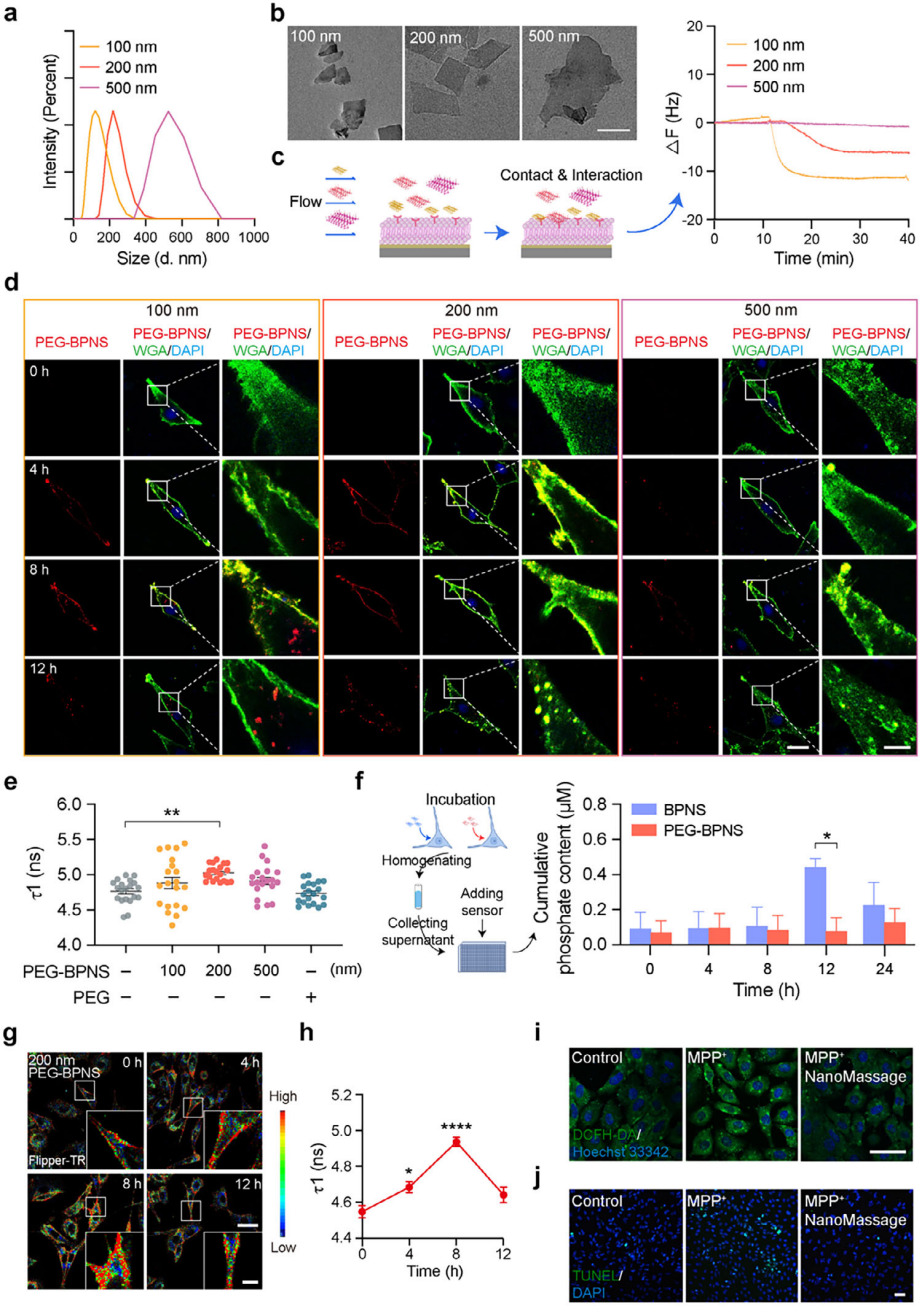
NanoMassage increase PM tension of neuron‐like cells, preventing MPP^+^‐induced cytotoxicity on cells. a) Size distribution of PEG‐BPNS with gradient sizes in deionized water. b) Transmission electron microscopy (TEM) images of PEG‐BPNS with gradient sizes. Scale bar, 200 nm. c) Quartz crystal microbalance with dissipation (QCM‐D) experiment capturing the interactions between the neural cell membrane and PEG‐BPNS with gradient sizes, respectively. Briefly, PEG‐BPNS with gradient sizes flowed across an Au chip spin‐coated with the cell membrane of SH‐SY5Y to assess the interactions between the neural cell membrane and PEG‐BPNS. d) Observation of different sizes of PEG‐BPNS adhesion with the cell membrane of neural cells after incubation for indicated times, respectively. Confocal imaging indicates the adhesion and internalization of PEG‐BPNS (RdB, red) in neural cells. The cell membrane was labeled by Texas Red‐X Conjugated Wheat Germ Agglutinin (WGA, original color: red; false color: green), and cell nuclei were labeled with 4',6‐diamidino‐2‐phenylindole (DAPI) (blue). Scale bars: 20 µm (original images) and 5 µm (zoomed‐in images). e) 200 nm PEG‐BPNS adhesion results in the increased PM tension of SH‐SY5Y cells. SH‐SY5Y cells were incubated with different sizes of PEG‐BPNS for 8 h, and the lifetime of the Flipper‐TR probe expressing PM tension was measured by FLIM (*n* = 20 independent cells from three biological replicates). f) Cumulative phosphate release from 200 nm BPNS and PEG‐BPNS in neural cells for 0, 4, 8, 12, and 24 h, indicating 200 nm PEG‐BPNS prevent the sharp increase in the phosphate concentration of neural cells (*n* = 3 biological replicates). g) 200 nm PEG‐BPNS increased PM tension of neuron‐like cells after incubation for 8 h. Neuron‐like cells were treated with 200 nm PEG‐BPNS for indicated times, and the lifetime of the Flipper‐TR probe reflecting PM tension was observed by FLIM. Scale bars: 20 µm (original images) and 5 µm (zoomed‐in images). h) Quantitative analysis of panel (g). *n* = 3 biological replicates. i) Representative fluorescence images of intracellular ROS levels (stained by DCFH‐DA probe, green) in neuron‐like cells with indicated treatments. Scale bar, 50 µm. j) TUNEL assay of neuron‐like cells with indicated treatments. Scale bar, 50 µm. Data are shown as means ± standard error of the mean (SEM). Statistical significance was determined by e) one‐way analysis of variance (ANOVA) with a Dunnett's multiple comparisons test and h) a Šídák's multiple comparisons test or f) by two‐way ANOVA with a Šídák's multiple comparisons test. *p < 0.05, **p < 0.01, and ****p < 0.0001.

Investigations into 2D nanosheets have revealed size‐dependent interactions with the cell membrane. Smaller nanosheets tend to penetrate the cell membrane, while larger nanosheets have a propensity to adhere to the cell membrane.^[^ ^18^, ^24^ ^]^ Furthermore, according to our previous study, PEGylated nanosheets could form a special nano–bio interface with the neural cell membrane to generate therapeutic effects in disease.^[^ ^18c^ ^]^ We thus synthesized PEG‐BPNS with average sizes of ≈100, ≈200, and ≈500 nm to identify the optimal size inducing extracellular forces (**Figure** **1**a). Transmission electron microscopy (TEM) analysis revealed typical sheet‐like morphology for PEG‐BPNS with varying sizes (Figure 1b). Additionally, the modification of PEG motif on BPNS was confirmed through analyzing surface zeta potential (Figure S1a, Supporting Information) and Fourier transform‐infrared spectroscopy (FTIR) analyses (Figure S1b–d, Supporting Information). Notably, the surface chemistry of PEG‐BPNS, determined by PEG density, is uniform across different sizes, as indicated by the similar surface zeta potential among PEG‐BPNS of varying dimensions. Collectively, these results suggest the successful preparation of PEG‐BPNS with comparable morphology and surface chemistry but distinctive dimensions.

The effectiveness of “NanoMassage” in producing forces to cell membranes is dependent on two main factors, including the amount of nanosheets interacting with cell membranes and the duration of dynamical interaction between nanosheets and cell membranes. To investigate the size‐dependent interactions of PEG‐BPNS with various dimensions in neural cells, we used a quartz crystal microbalance (QCM) assay to assess the interactions between PEG‐BPNS and membranes from cultured SH‐SY5Y neural cells (Figure 1c). Downward frequency curves indicated contact and adsorption of PEG‐BPNS onto the cell membrane. The amount of PEG‐BPNS adhering to the cell membrane varied with sizes, with 100, 200, and 500 nm PEG‐BPNS showing decreasing adherence in that order. Subsequently, we proceeded to observe the duration of interaction between PEG‐BPNS and the PM of SH‐SY5Y neural cells. Determining the maximum usable concentration of PEG‐BPNS of various sizes that did not lead to a significant decrease in cell viability as 0.5 µg mL^−1^ (Figure S2, Supporting Information), we incubated cells with this concentration of PEG‐BPNS of different dimensions for varying periods. As shown in Figure 1d, during 12 h incubation of PEG‐BPNS, the amount of PEG‐BPNS adhering to the PM of neural cells increased gradually from 0 to 8 h, as indicated by the intensity of the fluorescent signal (red) on the cell membrane. The fluorescence intensity of PEG‐BPNS of different sizes on the cell surface was ranked highest for 100 nm, followed by 200 nm, and then 500 nm, which aligned with the previous QCM data. However, when the incubation time was extended to 12 h, the fluorescence signal of the 100 nm PEG‐BPNS was predominantly observed within the cells, suggesting that 100 nm PEG‐BPNS could penetrate the cell membrane and accumulate into the cells. Consequently, it can be inferred that the interaction between the 100 nm PEG‐BPNS and the PM of cells was briefer compared to that of other sizes. In essence, among the varying sizes of PEG‐BPNS, the larger amount of 200 nm PEG‐BPNS adhered to the cell membrane and demonstrated a more prolonged interaction with the cell membrane.

Inspired by our previous study,^[^ ^18c^ ^]^ which has computationally constructed interaction modes for PEGylated nanosheets and cell membranes, and verified that the effective interaction of PEGylated nanosheets could be extended to various 2D nanomaterials such as graphene oxide and poly(l‐lactide acid) nanosheets, we next investigate whether the adhesive force produced by PEG‐BPNS changes membrane tension and thus modulates intracellular processes.^[^ ^25^ ^]^ We initially gauged the impact of PEG‐BPNS with various dimensions on the membrane tension of neural cells after an 8 h incubation using the recently developed mechanosensitive probe “fluorescent lipid tension reporter” (Flipper‐TR), which consists of twisted push–pull fluorophore that could stably integrate into the PM of cells where its planarization and polarization, and thus sensitize to mechanical forces acting on the membrane.^[^ ^26^ ^]^ Specifically, its fluorescence lifetime, which can be determined by fluorescence lifetime imaging microscopy (FLIM), changes linearly with PM tension in model membranes and mammalian cells, making it a powerful tool for monitoring PM tension changes in situ.^[^ ^27^ ^]^ As depicted in Figure 1e, it was observed that 200 nm PEG‐BPNS induced an increase in the Flipper‐TR lifetime when compared to the other experimental groups. Meanwhile, the Flipper‐TR lifetime was found to remain relatively unchanged in the PEG‐treated group, indicating that PM tension may not be solely dependent on the presence of the PEG motif. From these results above, we found that the variation in PM tension was directly linked to both the amount of PEG‐BPNS attached to the cell membrane and the duration of interaction between PEG‐BPNS and the cell membrane. Notably, there was no significant change in the level of cell membrane tension in the other groups, likely due to the shorter interaction time between 100 nm PEG‐BPNS and the cell membrane, as well as the lower quantity of 500 nm PEG‐BPNS adhering to the cell membrane.

Although BPNS are believed as biodegradable nanosheets to perform therapeutic effects in neurodegenerative disease,^[^ ^28^ ^]^ they also have the potential to induce programmed cell death by rapidly producing large amounts of phosphate anions.^[^ ^29^ ^]^ To avoid the cytotoxicity generated by PEG‐BPNS degradation, we investigated the levels of phosphate anions in cells treated with 200 nm PEG‐BPNS using a phosphate sensor (Figure 1f). The results revealed that incubation of PEG‐BPNS did not influence cellular phosphate anions during incubation for 24 h, while BPNS significantly elevated cellular phosphate anions after incubation for 12 h compared to PEG‐BPNS. Furthermore, to ensure 200 nm PEG‐BPNS enable harmless clearance from the body in a reasonable period after fulfilling their therapeutic functions, we investigated their biodegradation behavior in vitro by monitoring their absorbance. Four different physiological conditions were utilized, including air‐exposed deionized (DI) water, phosphate buffer saline (PBS), Dulbecco's modified eagle medium (DMEM) containing 10% fetal bovine serum (FBS), and artificial cerebrospinal fluid (aCSF) (Figure S3, Supporting Information). 200 nm PEG‐BPNS (20 ppm) were dispersed in different solutions at 37 °C for 96 h and then their absorbances were examined at predetermined time intervals (0, 12, 24, 48, 72, and 96 h) after centrifuging to replace the solutions with DI water. The UV–vis absorption of 200 nm PEG‐BPNS decreased significantly after 12 h, and the degree of decrease increased with time, indicating the biodegradability of 200 nm PEG‐BPNS. In conclusion, these findings provide evidence that 200 nm PEG‐BPNS could regulate the PM tension of neural cells by adhering to the cell membrane without causing changes in the level of cellular phosphate anions, thereby mitigating the cytotoxic effects associated with abrupt rises in phosphate anion concentrations.

Previous studies have shown that alterations in the biophysical characteristics of the cell membrane can impact the conformational changes of mechanosensitive receptors, leading to the transmission of mechanical signals and thereby influencing intracellular processes such as mitochondrial dynamics and metabolism.^[^ ^30^ ^]^ These interactions ultimately play a crucial role in determining cell fate during the development of diseases.^[^ ^31^ ^]^ We thus established an 1‐methyl‐4‐phenylpyridinium (MPP^+^)‐induced cell model exhibiting PD‐related damage phenotypes to verify the therapeutic effects of 200 nm PEG‐BPNS in vitro. Neural cells were differentiated into neuron‐like cells through a 72 h retinoic acid (RA) stimulation (Figure S4, Supporting Information), with subsequent induction of PD‐related damage phenotypes in neuron‐like cells through a 24 h exposure to MPP^+^. We initially confirmed the change of PM tension induced by 200 nm PEG‐BPNS in neuron‐like cells, as evidenced by a significantly enhanced lifetime of Flipper‐TR at 8 h of incubation (Figure 1g,h). These observations imply that 200 nm PEG‐BPNS are the identical PEG‐BPNS to generate massage‐mimicking effects in neuron‐like cells, characterized by their ability to 1) adhere to the cell membrane in significant quantities; 2) interact with the cell membrane over a duration of time; 3) prompt alterations in PM tension of neuron‐like cells; and 4) demonstrate excellent biodegradability and biocompatibility. Consequently, our study delves into the therapeutic potential of 200 nm PEG‐BPNS (noted by “NanoMassage”) in alleviating PD. MPP^+^ exerts its neurotoxic action on mitochondria to selectively damage neurons and to produce symptoms that are similar to those observed in PD, including oxidative stress and apoptosis.^[^ ^32^ ^]^ Therefore, we monitored the effects of NanoMassage on MPP^+^‐induced oxidative stress and apoptosis in neuron‐like cells. Oxidative stress was evaluated by monitoring intracellular reactive oxidative stress (ROS) levels using ROS‐sensitive 2′,7′‐dichlorofluorescein diacetate (DCFH‐DA) dye (Figure 1i). The DCFH‐DA signal was upregulated after exposure to MPP^+^, while NanoMassage downregulated the signal (Figure S5a, Supporting Information), implying that NanoMassage inhibit ROS generation. Since mitochondrial function is essential for cell survival and disruption can lead to apoptosis,^[^ ^33^ ^]^ we examined whether NanoMassage could mitigate apoptosis in PD model cells. The terminal deoxynucleotidyl transferase mediated deoxyuridine triphosphate (dUTP) nick‐end labeling (TUNEL) assay revealed that NanoMassage prevented the apoptosis of neuron‐like cells compared to MPP^+^‐induced cells (Figure 1j; Figure S5b, Supporting Information). These results collectively demonstrate that NanoMassage suppress ROS generation and prevent cellular apoptosis in PD model cells, suggesting their potential in PD treatment.

### 2.2 NanoMassage Restore the Movement Function and Alleviate PD‐Related Neuropathology

**Figure 2:**
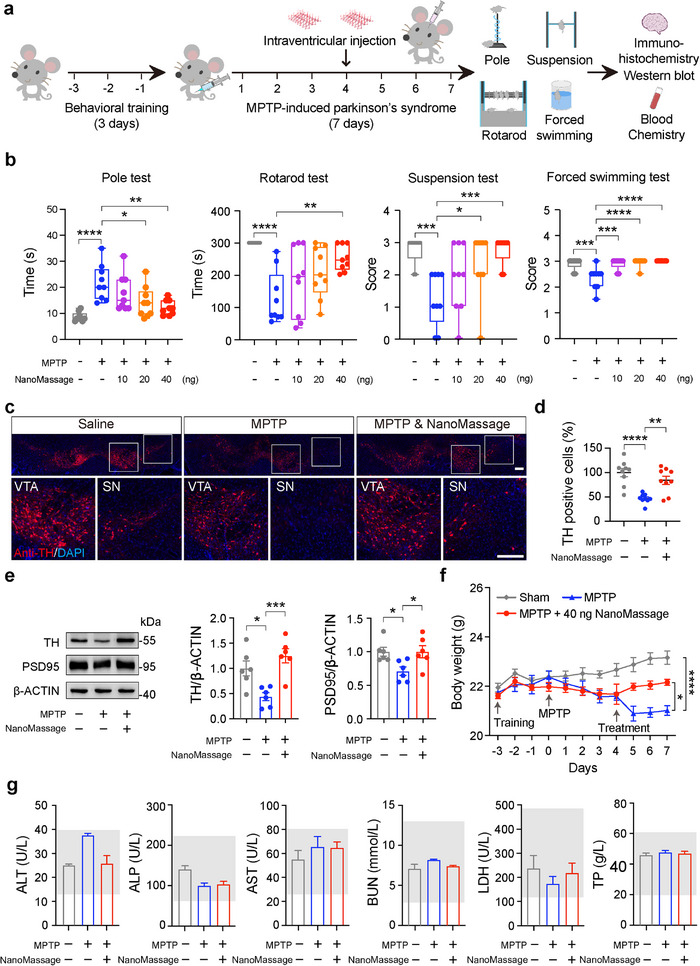
NanoMassage effectively alleviate PD with undetectable toxicity. a) Treatment schedule of MPTP‐induced mouse model of PD in the presence or absence of NanoMassage. Mice were behaviorally trained for 3 days prior to producing experimental Parkinsonism by MPTP. MPTP was administered intraperitoneally for a continuous period of 7 days (25 mg kg^−1^, once a day) to induce the disease model. Mice were given an intracerebroventricular injection on the 4^th^ day of MPTP administration, including one of the following treatments: 10 ng per mouse of NanoMassage, 20 ng per mouse of NanoMassage, 40 ng per mouse of NanoMassage or equal volume saline. On the 8^th^ day, mice were sacrificed and dissected after four different behavioral tests as the schematic shows. Mice from each group were randomly divided into two subgroups: three mice for immunohistochemistry and six mice for western blot. b) Behavior analysis of pole test, rotarod test, suspension test, and forced swimming test after administration of NanoMassage or saline (*n* = 9 independent mice per group). c) Representative fluorescence images of dopaminergic neurons (anti‐TH, green) in both the SN and the VTA of mice. Cell nuclei were stained with DAPI (blue). Scale bars, 200 µm. d) Graph shows the percentages of TH‐positive neurons in both the SN and the VTA of mice. *n* = 9 independent fields from three mice per group. e) Representative western blot images and quantification of TH and PSD95 proteins in the SN region from mice (*n* = 6 independent mice per group). Quantification of TH and PSD95 levels normalized to β‐ACTIN levels. f) Body weights of mice with different administrations (*n* = 9 independent mice per group). g) Detection of biochemical indicators of animal serum in different treatment groups (*n* = 3 independent mice per group). ALT: alanine aminotransferase, ALP: alkaline phosphatase, AST: aspartate aminotransferase, BUN: blood urea nitrogen, LDH: lactate dehydrogenase, TP: total protein. Data are shown as means ± SEM. Statistical significance was determined by one‐way ANOVA with b,d,e) a Dunnett's multiple comparisons test and g) a Šídák's multiple comparison test or by f) two‐way ANOVA with a Tukey's multiple comparisons test. *p < 0.05, **p < 0.01, ***p < 0.001, and ****p < 0.0001.

In light of the neuroprotective effect of NanoMassage in vitro, we constructed the PD mouse model to investigate the effects of NanoMassage in modulating motor coordination and attenuating neuropathology in vivo. Prior to model induction, mice were trained for 3 days to adapt to four different behavioral tests that we used to evaluate motor coordination. Subsequently, MPTP was injected intraperitoneally for 7 days, while NanoMassage were singly injected into the ventricle by microtargeted intracerebral injection on the 4^th^ day of administration. On the 8^th^ day, the motor coordination ability of mice in each group was evaluated via pole, rotarod, suspension, and forced swimming tests (**Figure** **2**a). To investigate the dose‐dependent effects of the NanoMassage, we administered doses of 10, 20, and 40 ng per mouse of NanoMassage to MPTP‐induced PD mice, respectively. As shown in Figure 2b, compared to healthy mice, PD mice showed an increased dwelling time in the pole, which was attributed to neurotoxicity caused by MPTP. On the other hand, PD mice treated with NanoMassage showed a reduced dwelling time, indicating that NanoMassage significantly improve the locomotor behavior of PD mice. Similar results were obtained by the rotarod test and the suspension test. In comparison with healthy mice, PD mice exhibited motor deficits in the latency to fall on a rotarod and the reduced score in suspension; in contrast, NanoMassage‐treated PD mice demonstrated a prolonged run duration time on a rotarod and increased score in suspension, suggesting that NanoMassage enhance the motor coordination of PD mice. In the forced swimming test, an anxiety‐related behavioral test, NanoMassage administration elevated the score of the swimming condition of mice, suggesting the antianxiety effect of NanoMassage. Moreover, the improvement of NanoMassage on the behavioral performance of PD mice was found to be dose dependent (Figure 2b). The administration of 40 ng NanoMassage elevated the scores to a level comparable to that of nonmodel mice in the suspension test and the forced swimming test, indicating the superior therapeutic efficacy of NanoMassage in PD.

PD is characterized by dopaminergic neuronal dysfunction in the SN region, as demonstrated by decreased levels of tyrosine hydroxylase (TH)—an enzyme involved in dopamine synthesis and postsynaptic density (PSD)—a protein associated with synaptic plasticity.^[^ ^34^ ^]^ To evaluate the effects of NanoMassage on dopaminergic neuronal function in PD mice, TH immunohistochemistry was performed (Figure 2c). Mice treated with 40 ng of NanoMassage exhibited a higher number of TH‐positive neurons in both the SN and the ventral tegmental area (VTA) compared to the MPTP group (Figure 2d), indicating that NanoMassage significantly mitigated MPTP‐induced dopaminergic neuron loss. Additionally, we analyzed TH and PSD expression levels in the SN. The higher expressions of TH and PSD95 were observed in the SN from PD mice administrated with 40 ng of NanoMassage in comparison with saline‐treated PD mice (Figure 2e), proving that NanoMassage restore the neuronal functions in mouse model of PD. Collectively, these behavioral and neuroanatomical results from the MPTP‐induced PD mice demonstrated that NanoMassage protected against the loss of motor coordination and dysfunction of dopaminergic neurons which occurred in PD. Furthermore, we evaluated the biological safety of NanoMassage by monitoring changes in mouse body weight during administration and assessing liver and kidney function. The weight of mice treated with MPTP decreased significantly, while NanoMassage administration gradually regained the weight to a normal level (Figure 2f). To examine the safety of NanoMassage in vivo, the serum biochemical tests and several important hepatic and kidney function parameters were measured, including alanine aminotransferase (ALT), alkaline phosphatase (ALP), aspartate aminotransferase (AST), lactate dehydrogenase (LDH), total protein (TP), and blood urea nitrogen (BUN). These parameters were all in the normal range and showed no distinct changes after NanoMassage administration (Figure 2g), suggesting unaffected hepatic and kidney functions.

To confirm that the protective effect on dopaminergic neurons was mediated by NanoMassage, mice were administered 40 ng of RdB/NanoMassage via intraventricular injection on the 4^th^ day of MPTP administration (Figure S6a, Supporting Information). Motor coordination was then assessed 24 h after RdB/NanoMassage administration. As shown in Figure S6b (Supporting Information), 40 ng of RdB/NanoMassage significantly improved the locomotor behavior of PD mice. Following this, mice were sacrificed and dissected for TH immunohistochemistry (Figure S6c, Supporting Information). Co‐localization of RdB/NanoMassage fluorescence signals with TH staining confirmed the presence of NanoMassage within dopaminergic neurons. Mice treated with NanoMassage showed a greater number of TH‐positive neurons in the SN compared to the MPTP group, further confirming that the protective effect on dopaminergic neurons was mediated by NanoMassage.

### 2.3 NanoMassage Induce the Reorganization of Actin Cytoskeleton through RhoA/ROCK Axis

**Figure 3:**
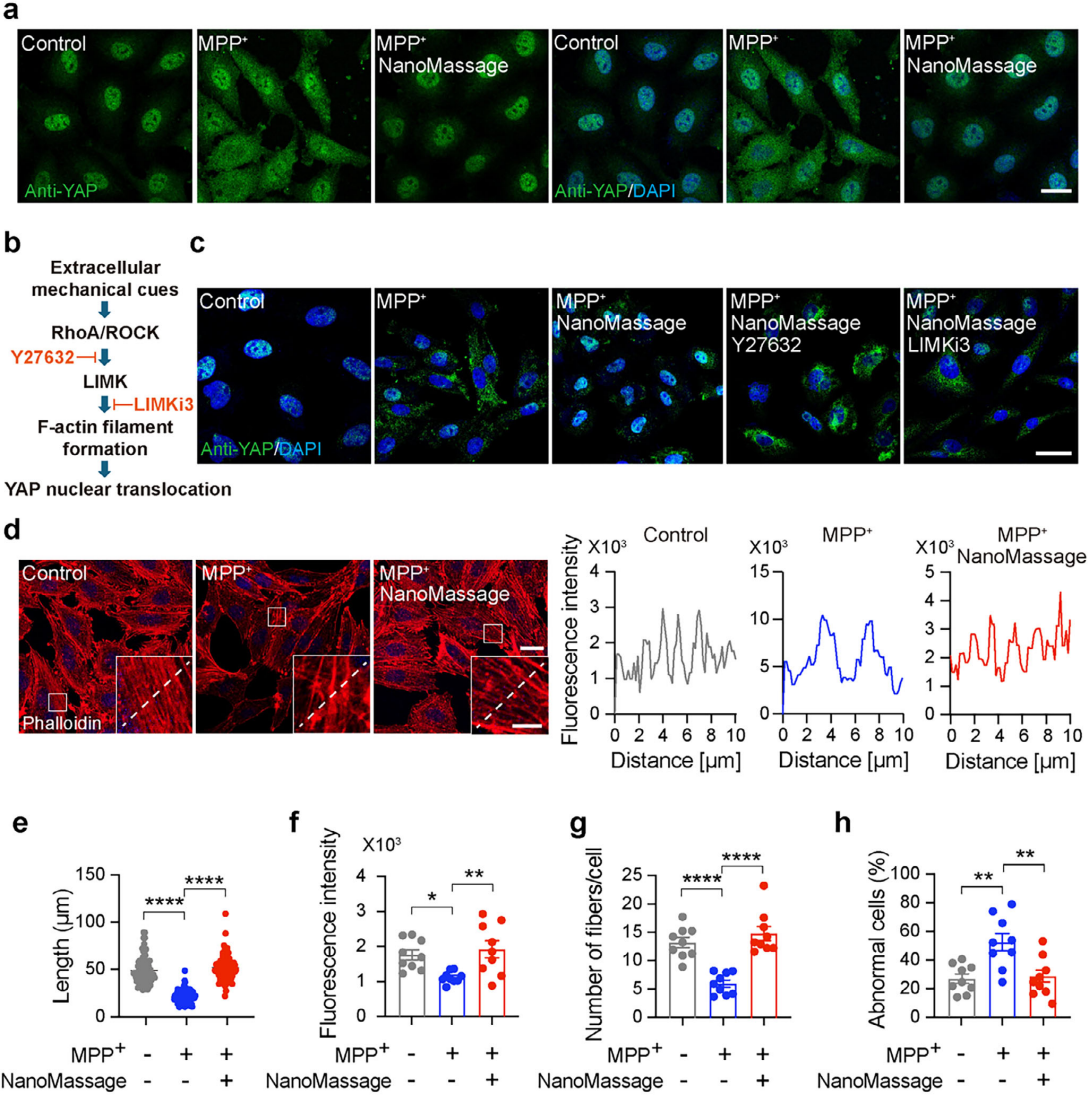
NanoMassage induce the reorganization of actin cytoskeleton through RhoA/ROCK axis in neuron‐like cells. a) Confocal immunofluorescence images showing YAP subcellular localization in neuron‐like cells. Scale bar, 20 µm. b) Schematic representation of the proposed interplay between Rho/ROCK pathway and YAP nuclear shuttling, as demonstrated by selective inhibitors of Rho/ROCK pathway components. c) Confocal representative images of neuron‐like cells with indicated treatments and stained with anti‐YAP (green). Cell nuclei were labeled with DAPI (blue). Scale bar, 20 µm. d) NanoMassage dramatically rescue the disordered actin cytoskeleton caused by MPP^+^. Left: confocal images showing changes of F‐actin in neuron‐like cells with indicated treatments. Neuron‐like cells were stimulated with MPP^+^ for 16 h, and then treated with or without NanoMassage for 8 h. Phalloidin (original color: far red; false color: red) was used for F‐actin labeling and DAPI (blue) was used for cell nuclei labeling. Scale bars: 20 µm (original images) and 5 µm (zoomed‐in images). Right: the fluorescence distribution of actin at the white dashed line. e) Statistical analysis of microfilament lengths in each condition as in panel (d). *n* = 40 biologically independent cells from three independent experiments. Statistical analysis of f) microfilament fluorescence intensity, g) number of fibers per cell, and h) cells with disordered F‐actin structures in each condition as in panel (d) (*n* = 9 fields from three biological replicates). Data are shown as means ± SEM. Statistical significance was determined by one‐way ANOVA with g) a Šídák's multiple comparisons test and e,f,h) Dunnett's multiple comparisons test. *p < 0.05, **p < 0.01, and ****p < 0.0001.

We next elucidate the mechanism by which NanoMassage alleviate PD. Given the ability of NanoMassage to apply mechanical stimulation to neuron‐like cells for ameliorating PD‐related symptoms, we examined the primary effector that responds to such stimulation within cells. The yes‐associated protein (YAP) is a mechanically sensitive transcriptional activator that is sensitive to mechanical cues, such as substrate adhesion and tensile forces, and plays a critical role in tissue and organ development, remodeling, and various diseases.^[^ ^35^ ^]^ The function of YAP is tightly dependent on translocation from the cytoplasm to the nucleus in response to diverse physical cues. Increased mechanical tension promotes YAP nuclear localization and transcriptional activity, influencing downstream pathways, including Wnt/β‐Catenin pathway, transforming growth factor‐β (TGF‐β)‐mediated responses and phosphatidylin‐ositol‐3‐kinase (PI3K)–protein kinase B (AKT)–mammalian target of rapamycin (mTOR) signaling pathway to regulate critical cellular functions and tissue homeostasis.^[^ ^36^ ^]^ As the activity of YAP is closely linked to its subcellular localization, being activated in the nucleus and inhibited in the cytoplasm,^[^ ^37^ ^]^ we observed the subcellular localization of YAP in cells treated with NanoMassage. NanoMassage‐mediated mechanical stimulation led to YAP nuclear translocalization in cells (**Figure** **3**a), promoting the activation of YAP in PD model cells.

Given that the RhoA/ROCK axis is widely recognized as a primary mechanosensing pathway responsible for perceiving mechanical stimuli and modulating YAP translocation and actin polymerization,^[^ ^38^ ^]^ we sought to determine whether the effects of NanoMassage on YAP translocation are mediated through this pathway. To investigate this, cells were treated with pharmacological inhibitors targeting the Ras homolog gene family member A (RhoA)/Rho‐associated kinase (ROCK) axis—Y27362 (a ROCK inhibitor) and LIMKi3 (a LIM kinase inhibitor)^[^ ^38^ ^]^ (Figure 3b). These inhibitors were employed to disrupt RhoA/ROCK signaling and evaluate their impact on NanoMassage‐induced YAP localization. Our results demonstrated that treatment with Y27362 and LIMKi3 induced the cytoplasmic shuttling of YAP, effectively abolishing the NanoMassage‐induced nuclear translocation of YAP (Figure 3c). These findings indicate that the RhoA/ROCK axis is essential for mediating the mechanical stimuli generated by NanoMassage to regulate YAP translocation.

YAP nuclear import requires a mechanical connection between the cytoskeletal dynamics and the nucleus;^[^ ^35^, ^39^ ^]^ we next carefully examined the alterations in the actin cytoskeleton of MPP^+^‐induced neuron‐like cells after being treated with NanoMassage for 8 h, utilizing phalloidin staining for F‐actin visualization. In the control group, the actin cytoskeleton exhibited a homogeneous and orderly distribution, whereas MPP^+^ induction led to disturbed distribution of F‐actin (Figure 3d). Moreover, MPP^+^ induction resulted in a significant decrease in length (Figure 3e), mean intensity (Figure 3f), and number of F‐actin (Figure 3g) compared to the control group. The MPP^+^‐induced neuron‐like cell group also exhibited an increased presence of cells with abnormal actin filaments (Figure 3h). Conversely, the homeostasis of the actin filaments after NanoMassage treatment displayed a trend toward restoration, indicating that NanoMassage promote the depolymerization of actin filaments in MPP^+^‐inductive cells. The polymerization and depolymerization of actin filaments can modulate membrane constriction at organelle contact sites and regulate the biogenesis and dynamics of organelles. These findings suggest that NanoMassage can generate mechanical stimuli that leads to the activation of YAP and subsequently regulates the depolymerization of actin filaments in PD model cells.

### 2.4 NanoMassage Regulate Inter‐Organelle Contacts to Protect Against MPP^+^‐Induced Mitochondrial Dysfunction

**Figure 4:**
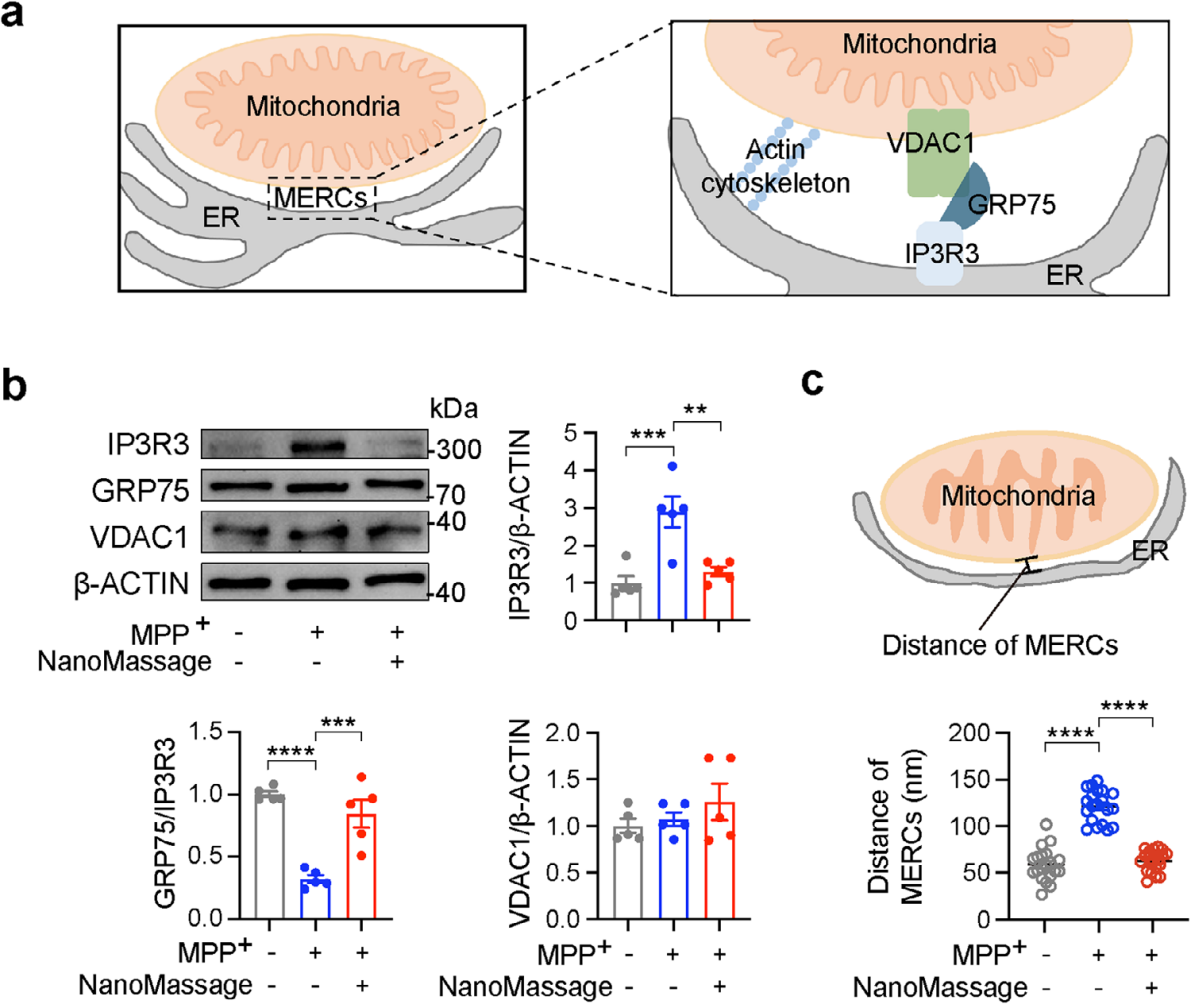
NanoMassage modulate MERCs in neuron‐like cells. a) Schematic illustration of the location of actin cytoskeleton and IP3R3–GRP75–VDAC1 complex at MERCs. b) Western blot analysis and quantification of IP3R3, GRP75, and VDAC1 expressions in neuron‐like cells with different treatments. *n* = 5 biological replicates. The values were normalized to the control group. c) Quantification of MERCs in neuron‐like cells with indicated treatments (*n* = 20). Data are shown as means ± SEM. Statistical significance was determined by one‐way ANOVA with a Dunnett's multiple comparisons test. **p < 0.01, ***p < 0.001, and ****p < 0.0001.

The actin cytoskeleton is widely recognized to play a pivotal role in the formation and regulation of MERCs, which are critical for modulating mitochondrial dynamics, including fission and fusion processes.^[^ ^4^ ^]^ At MERCs, the ER and mitochondria are physically interconnected through protein tethers, such as the IP3R3–GRP75–VDAC1 complex^[^ ^40^ ^]^ (**Figure** **4**a). The polymerization and depolymerization of actin filaments have been shown to influence the clustering and functional activity of IP3R3, thereby regulating the dynamics of the IP3R3–GRP75–VDAC1 complex and MERCs functionality.^[^ ^41^ ^]^ To explore the potential of NanoMassage in modulating MERCs, we assessed the expression levels of IP3R3, GRP75, and VDAC1 in an MPP^+^‐induced cell model of PD. Immunoblotting analysis demonstrated that MPP^+^ treatment significantly upregulated IP3R3 expression and increased the GRP75/IP3R3 ratio, indicative of impaired MERC integrity. Notably, NanoMassage treatment attenuated the MPP^+^‐induced elevation in IP3R3 levels and restored the GRP75/IP3R3 ratio to baseline levels (Figure 4b). These results suggest that NanoMassage function as a modulator of MERCs by regulating the dynamics of the IP3R3–GRP75–VDAC1 complex. Additionally, we analyzed the MERCs using TEM to verify the modulation of NanoMassage on MERCs (Figure 4c). In an MPP^+^‐induced cell model of PD, we observed a significant increase in the average distance between mitochondria and the juxtaposing ER, indicative of disrupted MERCs. However, NanoMassage treatment effectively rescued MERCs, as evidenced by a significant reduction in the mitochondria–ER distance. These findings confirm the effect of NanoMassage on promoting MERCs’ formation, further supporting the potential of them to regulate mitochondrial dynamics.

**Figure 5:**
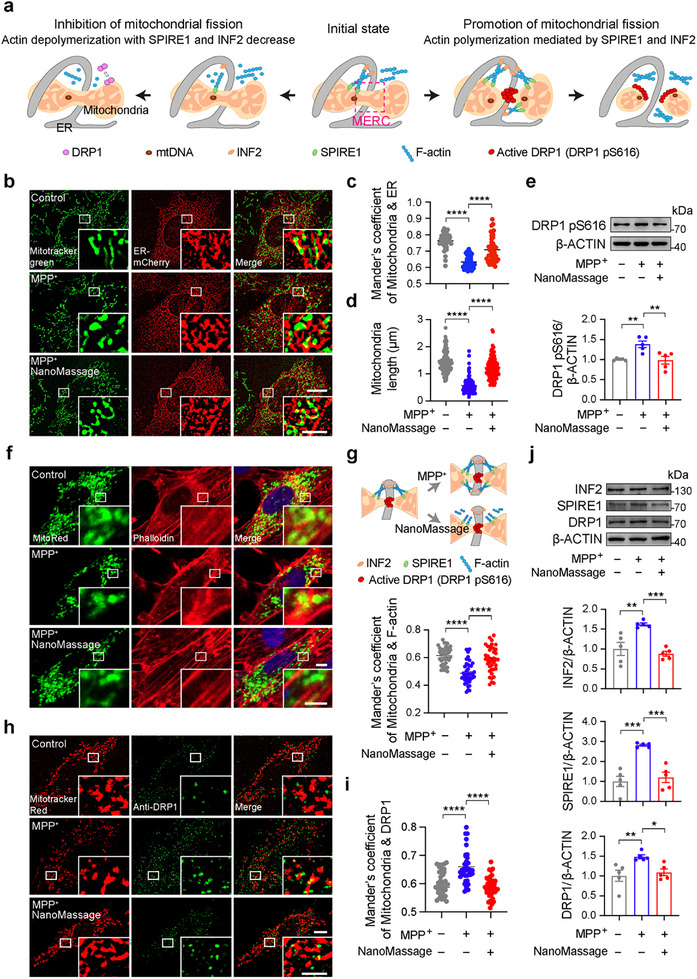
NanoMassage regulate inter‐organelle contacts to inhibit MPP^+^‐induced mitochondrial fission. a) Role of actin filaments in the process of mitochondrial fission. In the initial phases, actin‐nucleating proteins, including INF2 and SPIRE1, localize to ER and mitochondria, respectively. Subsequently, SPIRE1 interacts with INF2 to facilitate actin assembly on the surfaces of mitochondria, thereby establishing a link between mitochondria, the actin cytoskeleton, and the ER. Actin polymerization recruits phosphorylated DRP1 (DRP1 pS616) to mitochondria and promotes its oligomerization, generating a contractile force that drives mitochondrial division. Conversely, actin depolymerization inhibits the interaction between SPIRE1 and INF2, resulting in decreased levels of these actin‐nucleating proteins and consequently impeding mitochondrial fission. b) Representative structure illumination microscopy (SIM) images of co‐localization between mitochondria and ER in ER–mCherry (red) transfected neuron‐like cells with different treatments. Mitochondria were marked with Mitotracker (green). Scale bars: 10 µm (original images) and 2.5 µm (zoomed‐in images). c) Quantification of the co‐localization of mitochondria and ER in panel (b), indicating that NanoMassage treatment restores the co‐localization of mitochondria with ER in MPP^+^‐induced neuron‐like cells. *n* = 42 biologically independent cells from three independent experiments. d) Mitochondrial lengths were calculated in each condition as in panel (b). NanoMassage prevent MPP^+^‐induced mitochondrial fragmentation in neuron‐like cells. *n *= 120 biologically independent cells from three independent experiments. e) Western blot analysis and quantification of phosphorylated DRP1 (pS616) expression in neuron‐like cells with different treatments. *n* = 5 biological replicates. The values were normalized to the control group. f) Confocal microscopy image of F‐actin (Phalloidin) localization to a subset of mitochondria in neuron‐like cells transfected with mitochondrial red fluorescent protein (MitoRed). DAPI (blue) was used for cell nuclei labeling. Scale bars: 5 µm (original images) and 2 µm (zoomed‐in images). g) Quantification of the co‐localization of mitochondria and F‐actin in panel (f). Mander's of mitochondrial coefficients are shown for each condition. *n* = 40 cells from three biological replicates. h) Representative confocal images of co‐localization between endogenous DRP1 puncta and mitochondria in neuron‐like cells with different treatments. DRP1 and mitochondria were marked by anti‐DRP1 antibody (green) and Mitotracker red (red), respectively. Scale bars: 5 µm (original images); 2.5 µm (zoomed‐in images). i) Quantification of the co‐localization of mitochondria and DRP1 in panel (h). Mander's of mitochondria coefficients are shown for each condition. *n* = 20 cells from three biological replicates. j) Western blot analysis and quantification of INF2, SPIRE1, and DRP1 expressions in neuron‐like cells with different treatments. *n* = 5 biological replicates. The values were normalized to the control group. Data are shown as means ± SEM. Statistical significance was determined by one‐way ANOVA with a Dunnett's multiple comparisons test. *p < 0.05, **p < 0.01, ***p < 0.001, and ****p < 0.0001.

The modulation of mitochondrial dynamics at MERCs involves the coordinated activity of various proteins that regulate mitochondrial fission and fusion.^[^ ^4^, ^42^ ^]^ In the initial stages of mitochondrial fission, ER tubules delineate preconstriction sites at the contact sites between ER and mitochondria. Subsequently, ER‐associated INF2 promotes actin filament polymerization, while splice isoform of SPIRE aids in the assembly of actin on the mitochondria. The interaction between SPIRE1 and INF2 serves to connect the actin filaments, mitochondria, and ER. Assembly of actin filaments on mitochondria leads to the recruitment and oligomerization of phosphorylated DRP1 at the preconstriction sites, culminating in the mitochondrial fission process, followed by the disassembly of the fission complex and reduction of mitochondrial length (**Figure** **5**a). Conversely, the depolymerization of actin filaments inhibits the binding and oligomerization of phosphorylated DRP1 at the preconstriction site, thereby preventing the constriction and division of mitochondria. This is accompanied by a decrease in the expressions of INF2, SPIRE1, and DRP1, and an extended interaction among actin filaments, mitochondria, and ER.

We conduct experiments to investigate whether the effect of NanoMassage on ameliorating PD symptoms is mediated by reorganizing inter‐organelle contacts and ultimately impacting the dynamic of mitochondrial fission. The early events of both changes in ER–mitochondria contacts and mitochondrial length in different groups were checked. Compared to the MPP^+^ group, we observed an increase in mitochondrial length, as well as augmented ER–mitochondria contact sites after NanoMassage treatment (Figure 5b–d), indicating that NanoMassage impede mitochondrial fission and regulate mitochondrial morphology in PD model cells. To corroborate this finding, we evaluated the expression of the key factor in mitochondrial division, the active form of DRP1, DRP1 with Ser616 phosphorylation.^[^ ^43^ ^]^ Immunoblotting experiments showed that NanoMassage treatment reduced the increased expression of phosphorylated DRP1 (DRP1 pS616) induced by MPP^+^ (Figure 5e).

To investigate whether the inhibitory effect of NanoMassage on DRP1 phosphorylation is required by actin depolymerization, we visualized the polymerization of the actin cytoskeleton along mitochondria. Cells were transiently transfected with MitoRed and labeled with Phalloidin for actin cytoskeleton visualization (Figure 5f). A substantial reduction in surface co‐localization between F‐actin and mitochondria was observed in PD model cells, but NanoMassage treatment led to a significant increase in co‐localization, which is consistent with the impact of NanoMassage on regulating ER–mitochondria contacts (Figure 5g). The results demonstrated that NanoMassage treatment slowed down the fission of mitochondria by decelerating the polymerization of actin filaments on mitochondria, corresponding to the prolonged interaction between actin filaments and mitochondria. Considering that actin filaments’ polymerization induces assembly of DRP1 on the mitochondria, causing constriction and division of mitochondria,^[^ ^44^ ^]^ we then visualized the formation of DRP1 puncta at mitochondrial sites to examine the effect of NanoMassage on inhibiting DRP1 assembly (Figure 5h). As expected, in the group of MPP^+^‐induced cells, we observed an increased co‐localization between the endogenous DRP1 puncta and mitochondria compared to the control group (Figure 5i). In contrast, NanoMassage treatment revealed a noticeable reduction of the co‐localization between the endogenous DRP1 puncta and mitochondria, supporting our premise. Therefore, we then probed the level of relevant actin‐nucleating proteins in different groups to further substantiate the inhibition of NanoMassage on mitochondrial fission. As depicted in Figure 5j, cells treated with NanoMassage showed a significant decrease in INF2, SPIRE1, and DRP1 levels compared to MPP^+^‐induced cells, indicating that even if they do not enter the neuronal cells, NanoMassage still regulate the inter‐organelle contact and mitigate mitochondrial fission through inhibiting actin filaments polymerization‐induced DRP1 oligomerization on mitochondria in PD model cells.

Given that mitochondrial dynamics involve both fission and fusion processes and considering the role of the actin cytoskeleton in regulating mitochondrial fusion, we next examined the levels of OPA1 in MPP^+^‐induced cells treated with NanoMassage to elucidate their effects on mitochondrial fusion. As shown in Figure S7 (Supporting Information), OPA1 levels were significantly reduced in MPP^+^‐induced cells, consistent with impaired mitochondrial fusion. In contrast, NanoMassage treatment resulted in a notable increase in OPA1 levels, indicating that NanoMassage not only inhibit excessive mitochondrial fission but also promote mitochondrial fusion. These findings suggest that NanoMassage play a dual role in restoring mitochondrial dynamics, further supporting their therapeutic potential in mitigating mitochondrial dysfunction in PD. Considering that mitochondrial fragmentation is a known activator of mitophagy—a selective degradation pathway responsible for removing damaged or dysfunctional mitochondria through autophagy—this process plays a critical role in maintaining mitochondrial quality control and cellular homeostasis.^[^ ^45^ ^]^ We also examined the impact of NanoMassage on mitophagy in MPP^+^‐induced PD model cells. As shown in Figure S8 (Supporting Information), robust co‐localization of RFP‐LC3 puncta (representing autophagosomes) and Mitotracker‐labeled mitochondria were observed in MPP^+^‐induced PD model cells, indicating enhanced mitophagy. However, the co‐localization between mitochondria and autophagosomes was significantly reduced by NanoMassage treatment, consistent with a decrease in mitochondrial fragmentation. Collectively, these findings indicate that NanoMassage regulate MERCs to protect against MPP^+^‐induced mitochondrial dysfunction.

### 2.5 NanoMassage Have Vast Potential in Protecting Mitochondrial Functions under Mitochondrial Stress

Mitochondrial fission is the key regulator of the mitochondrial network and function. The flux of iterative fusion and fission events of mitochondria form mitochondrial dynamic networks, and healthy mitochondrial networks are essential for mitochondrial function and health. Mitochondrial dysfunction is strongly implicated in the pathogenesis of PD.^[^ ^46^ ^]^ Loss of mitochondrial membrane potential (△*ψ*_m_), indicative of early‐stage apoptosis, is intricately linked to mitochondrial dysfunction. To assess the impact of NanoMassage on the mitochondrial membrane potential, we employed 5,5′,6,6′‐tetrachloro‐1,1′,3,3′‐tetraethyl‐imidacarbocyanine iodide (JC‐1) to monitor mitochondrial depolarization. The red fluorescence of JC‐1 aggregates and the green fluorescence of JC‐1 monomers represent high and low △*ψ*_m_, respectively, with the green fluorescence indicating the early stage of apoptosis and the red fluorescence suggesting healthy cells.^[^ ^47^ ^]^ Exposure to MPP^+^ increased green fluorescent mitochondria compared with the control group (Figure S9, Supporting Information), indicating the dissipation of △*ψ*_m_. This phenomenon was also observed in other two traditional mitochondrial dysfunction cell models, which are produced by carbonyl cyanide *m*‐chlorophenyl hydrazone (CCCP) and oligomycin, respectively (Figure S10, Supporting Information). Conversely, cells with NanoMassage treatment exhibited strong red fluorescence akin to the control group, implying that NanoMassage treatment inhibits the loss of △*ψ*_m_. Analysis of the red/green fluorescence intensity ratio of JC‐1 staining confirmed NanoMassage attenuated the loss of △*ψ*_m_, indicating that NanoMassage could protect mitochondrial integrity in neuron‐like cells under mitochondrial stress.

To confirm the universality of NanoMassage in mitochondrial protection, we explored the therapeutic mechanism of NanoMassage in CCCP‐ and oligomycin‐induced cell models, respectively (Figure S11a,b, Supporting Information). Similar to the results of MPP^+^ induction, NanoMassage also effectively activated YAP and restored the disrupted distribution of F‐actin in neuron‐like cells subjected to CCCP and oligomycin. Therefore, we evaluated the mitochondrial network in the above neuron‐like cells subjected to PD‐related damage phenotypes (Figure S11c, Supporting Information). The staining of mitochondria with Mitotracker revealed that cells without any treatments exhibited a highly connected mitochondrial network, and it was difficult to distinguish single mitochondria. In contrast, cells induced by CCCP, oligomycin, and MPP^+^ produced fragmented mitochondrial networks with a greatly increased number of spherical mitochondria, which demonstrated that CCCP, oligomycin, and MPP^+^ caused a loss in network dynamics in cells. Treatment of NanoMassage led to marked mitochondrial fusion, resulting in an increase in the number of tubular mitochondria and a dense mitochondrial network. Moreover, NanoMassage decreased ROS production (Figure S12a,b, Supporting Information) and hindered cellular apoptosis (Figure S12c,d, Supporting Information) in CCCP and oligomycin‐induced cells. These results indicate that NanoMassage preserve mitochondrial integrity, restore mitochondrial networks, reduce oxidative stress, and prevent cellular apoptosis in the context of mitochondrial stress, highlighting the wide‐ranging potential of NanoMassage in protecting mitochondrial functions.

### 2.6 NanoMassage After Single Intranasal Administration Distribute in the Brain of PD Mice and Alleviate Their Movement Dysfunction and Neuropathology

**Figure 6:**
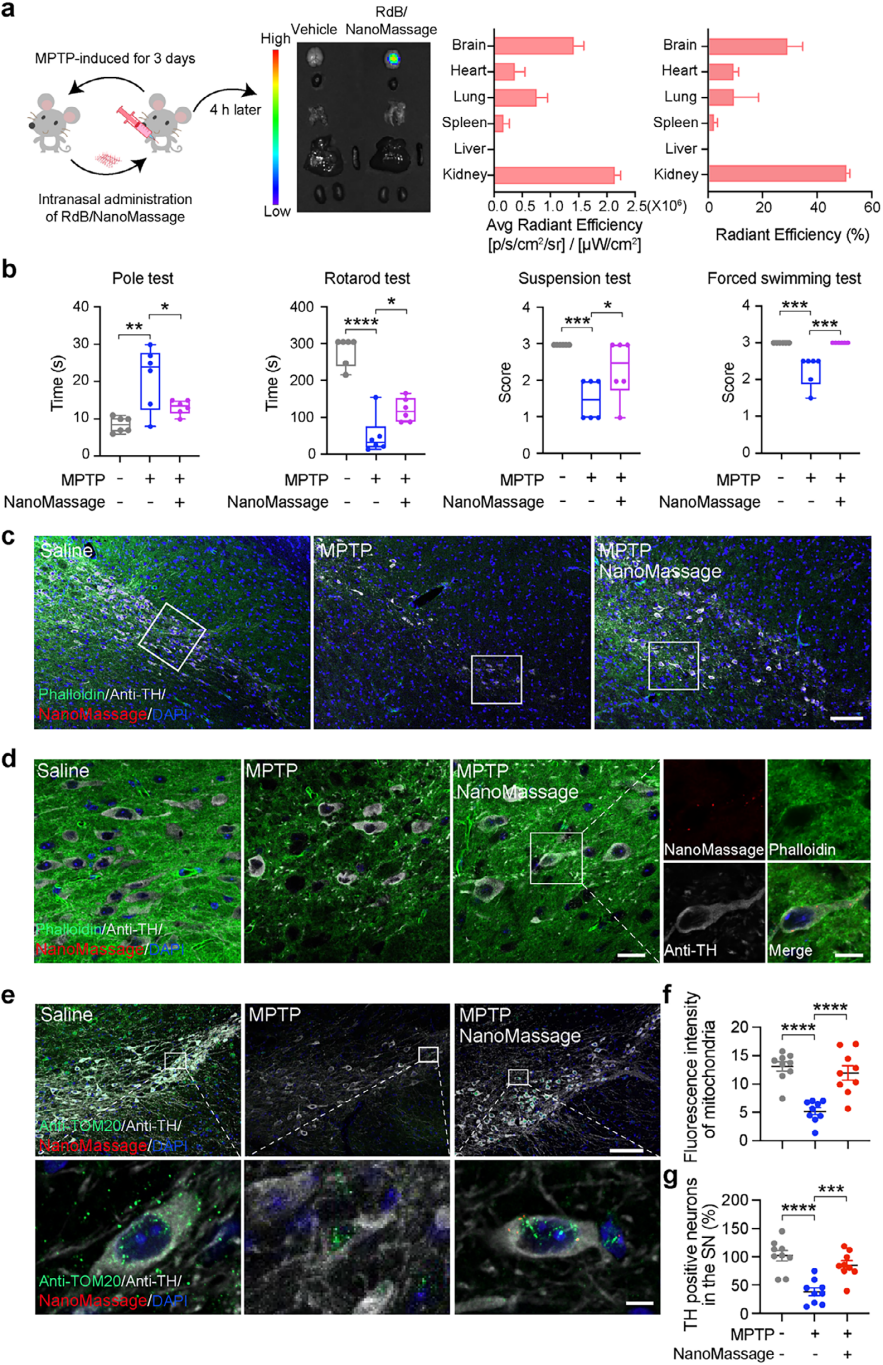
NanoMassage distribute in the brain of PD mice after a single intranasal administration and alleviate the motor dysfunction and neuropathology of mice. a) Schematic of workflow for intranasal administration of 0.1 mg kg^−1^ NanoMassage to mice with intraperitoneal injection of MPTP for 3 days (left). Representative fluorescence images indicating NanoMassage accumulate in mice brains after intranasal administration for 4 h (middle). The distribution of NanoMassage in the main organs of mice was observed and quantified with IVIS system (right). b) Behavior analysis of pole test, rotarod test, suspension test, and forced swimming test after intranasal administration of RdB/NanoMassage or saline for 24 h (*n* = 6 independent mice per group). c) Representative fluorescence images show the RdB/NanoMassage fluorescence signal (RdB, red), actin cytoskeleton (Phalloidin, original color: far red; false color: green), and dopaminergic neurons (anti‐TH, original color: green; false color: light dark) in the SN of mice. Cell nuclei were stained with DAPI (blue). Scale bar: 100 µm. d) Enlarged view of the area outlined by the rectangle in panel (c). Scale bars: 20 µm (original images) and 10 µm (zoomed‐in images). e) Representative fluorescence images show the RdB/NanoMassage fluorescence signal (RdB, red), mitochondria (anti‐TOM20, green), and dopaminergic neurons (anti‐TH, original color: far red; false color: light dark) in the SN of mice. Cell nuclei were stained with DAPI (blue). Scale bars: 100 µm (original images) and 5 µm (zoomed‐in images). f) Quantification of fluorescence intensity of mitochondria in the SN of mice. *n* = 9 independent fields from 3 mice per group. g) Graph shows the percentages of TH‐positive neurons in the SN of mice. *n* = 9 independent fields from 3 mice per group. Data are shown as means ± SEM. Statistical significance was determined by one‐way ANOVA with a Dunnett's multiple comparisons test. *p < 0.05, **p < 0.01, ***p < 0.001, and ****p < 0.0001.

To enhance the acceptability of the administration mode and achieve the massage‐mimicking effects noninvasively in deep brain regions, we evaluated the therapeutic effectiveness of intranasal administration of NanoMassage in PD mice. Intranasal administration, known for its noninvasiveness, enables swift brain targeting through the nasal route, presenting a contrast to direct intraventricular and intraparenchymal injections into brain tissue.^[^ ^48^ ^]^ Aside from drug molecules cleared via mucociliary processes, the residual drugs accessed the nasal cavity through both the neuronal pathway and systemic circulation. Considering previous findings demonstrating BPNS translocation across the nasal mucosal barrier,^[^ ^49^ ^]^ our current study seeks to assess the potential of NanoMassage bypassing the blood–brain barrier (BBB) through intranasal administration. RdB‐labeled NanoMassage (RdB/NanoMassage) were administered via intranasal administration at a dosage of 0.1 mg kg^−1^ to analyze the distribution of NanoMassage in MPTP‐induced PD mice. The main organs of mice were harvested for ex vivo IVIS imaging 4 h post administration. As depicted in **Figure** **6**a, the fluorescent signal of RdB/NanoMassage was clearly detected in mouse brains, indicating the distribution of NanoMassage in brain tissue. Quantification of the fluorescent signal of RdB/NanoMassage in main organs of mice revealed that more than 20% of the administered NanoMassage accumulated in the brain following intranasal administration, demonstrating their potential to target the SN and exert therapeutic effects.

Subsequently, we assessed the therapeutic efficacy of NanoMassage in PD mice through intranasal administration. PD mice model was induced as previously described, and RdB/NanoMassage were administered via intranasal administration at a dosage of 0.1 mg kg^−1^ on the 4^th^ day of MPTP administration. Behavioral patterns were further evaluated through the pole, rotarod, suspension, and forced swimming tests after intranasal RdB/NanoMassage administration for 24 h to assess the motor coordination of PD mice (Figure 6b). Successful model induction was confirmed by significant motor coordination deficits in MPTP model mice compared to healthy mice. Consistent with previous intra‐cerebroventricular injections, a single intranasal administration of NanoMassage displayed similar therapeutic efficacy in alleviating MPTP‐induced behavioral deficits in PD mice. In detail, PD mice with NanoMassage intranasal administration exhibited reduced time descending in the pole test, increased score in the forced swimming test, prolonged dwell time in the rotarod test, and decreased score in the suspension test, indicating improved motor functions of PD mice with NanoMassage treatment (Figure 6b). Moreover, no significant change in body weight was noted between PD mice treated with NanoMassage and saline‐treated PD mice during the 7 day MPTP treatment period (Figure S13, Supporting Information), indicating the biosafety of intranasal administration of NanoMassage.

To further investigate whether the therapeutic effects of NanoMassage in PD are mediated by actin cytoskeleton reorganization and mitochondrial protection, brain sections from MPTP‐induced PD mice were stained after RdB/NanoMassage treatment for 24 h (Figure 6c,d). As shown in the enlarged view (Figure 6d), MPTP‐induced mice displayed a reduced fluorescence intensity and distinct aggregation of actin cytoskeleton puncta, suggesting structural damage. In brain sections from PD mice treated with NanoMassage, distinct fluorescence signals of NanoMassage were observed in TH‐positive neurons, indicating effective delivery to the SN following intranasal administration. The increased fluorescence intensity and reduced aggregated puncta of the actin cytoskeleton in NanoMassage‐treated PD mice demonstrated that NanoMassage prevented MPTP‐induced damage to actin cytoskeleton integrity. Mitochondrial protection was assessed by staining SN sections with anti‐TOM20 (to mark a mitochondrial outer membrane protein) and anti‐TH antibodies (Figure 6e). In MPTP‐induced PD mice, mitochondria exhibited reduced fluorescence intensity and a perinuclear distribution, indicating elevated mitophagy and mitochondrial loss. However, treatment with NanoMassage significantly reversed these changes (Figure 6f), underscoring the protective effect of NanoMassage on mitochondrial integrity. Furthermore, quantification of TH‐positive neurons in the SN showed that a single intranasal NanoMassage administration provided dopaminergic neuron protection comparable to intra‐cerebroventricular injections (Figure 6g). In summary, NanoMassage promote actin cytoskeleton reorganization and protect mitochondria, alleviating dopaminergic neuronal loss in PD mice.

**Figure 7:**
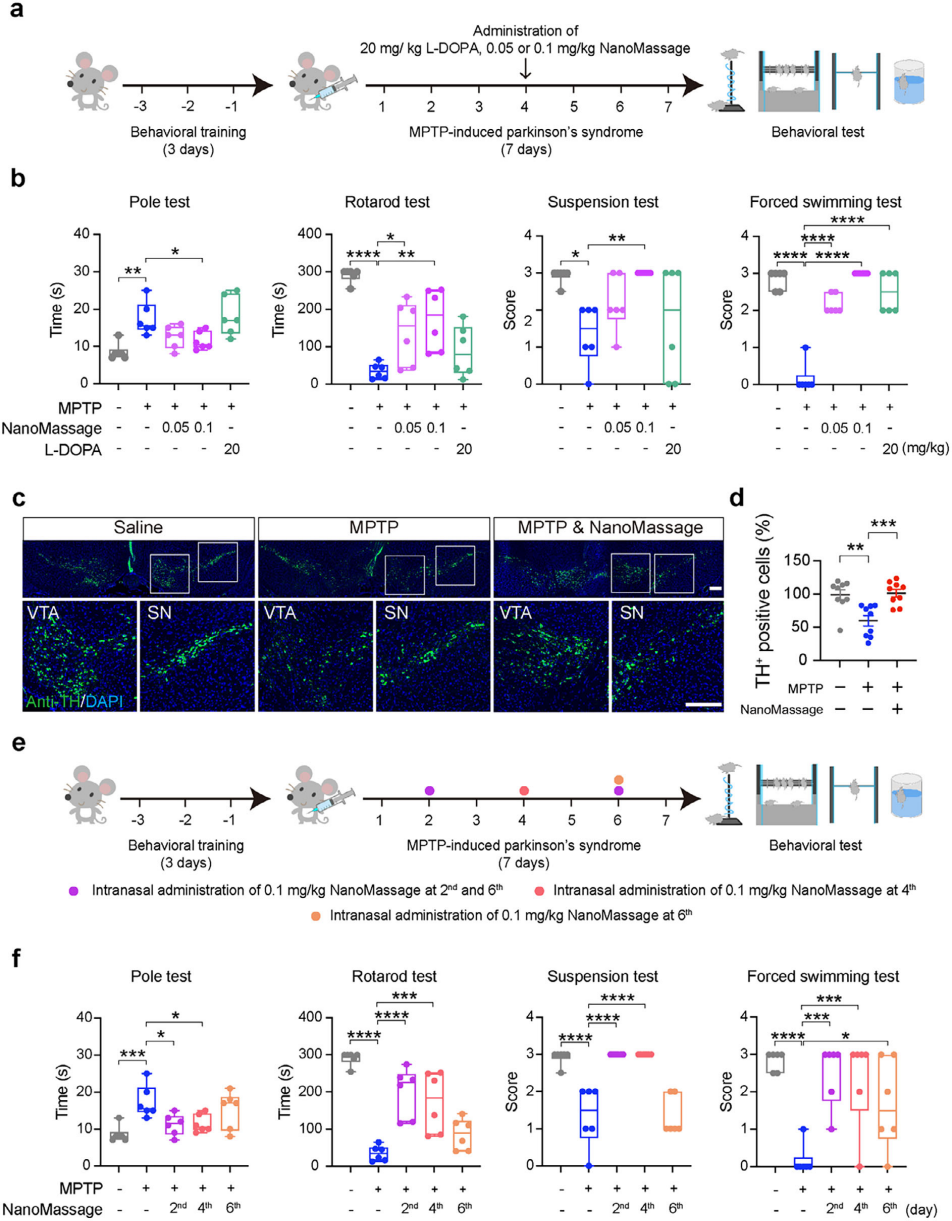
The therapeutic effects of intermediate doses or varied treatment schedules of NanoMassage in PD. a) Treatment schedule of MPTP‐induced mouse model of PD designed to compare the therapeutic effects of NanoMassage and L‐DOPA. Mice were behaviorally trained for 3 days prior to producing experimental Parkinsonism by MPTP. MPTP was administered intraperitoneally for a continuous period of 7 days (25 mg kg^−1^, once a day) to induce the disease model. Mice were given an oral administration of 20 mg kg^−1^ L‐DOPA, or an intranasal administration of 0.05 or 0.1 mg kg^−1^ NanoMassage on 4^th^ day of MPTP administration. On the 8^th^ day, mice were sacrificed and dissected after four different behavioral tests as the schematic shows. b) Behavior analysis of pole test, rotarod test, suspension test, and forced swimming test after administration of L‐DOPA, NanoMassage or saline (*n* = 6 independent mice per group). c) Representative fluorescence images of dopaminergic neurons (anti‐TH, green) in both the SN and the VTA of mice administered with 0.1 mg kg^−1^ NanoMassage or saline. Cell nuclei were stained with DAPI (blue). Scale bars, 200 µm. d) Graph shows the percentages of TH‐positive neurons in both the SN and the VTA of mice administered with 0.1 mg kg^−1^ NanoMassage or saline. *n* = 9 independent fields from 3 mice per group. e) Varied treatment schedules of NanoMassage in MPTP‐induced mouse model of PD. Mice were behaviorally trained for 3 days prior to producing experimental Parkinsonism by MPTP. MPTP was administered intraperitoneally for a continuous period of 7 days (25 mg kg^−1^, once a day) to induce the disease model. Mice were given an intranasal administration of 0.1 mg kg^−1^ NanoMassage on the indicated days of MPTP administration. On the 8^th^ day, mice were sacrificed and dissected after four different behavioral tests as the schematic shows. f) Behavior analysis of pole test, rotarod test, suspension test, and forced swimming test after administration of NanoMassage or saline (*n* = 6 independent mice per group). Data are shown as means ± SEM. Statistical significance was determined by one‐way ANOVA with a Dunnett's multiple comparisons test. *p < 0.05, **p < 0.01, ***p < 0.001, and ****p < 0.0001.

To assess the therapeutic efficacy of intranasally administered NanoMassage, we compared their effects on motor function improvement with those of levodopa (L‐DOPA), an oral medication widely used to compensate for dopamine depletion in PD progression.^[^ ^50^ ^]^ The PD mice model was induced as previously described, and NanoMassage were administered via intranasal administration at a low dosage of 0.05 or a high dosage of 0.1 mg kg^−1^ on the 4^th^ day of MPTP administration. Concurrently, L‐DOPA was administered orally at a dose of 20 mg kg^−1[^ ^51^ ^]^ as an additional control group. The therapeutic effects of NanoMassage and L‐DOPA were evaluated on day 8 using a series of behavioral tests, including the pole test, rotarod test, suspension test, and forced swimming test (**Figure** **7**a). As shown in Figure 7b, among these behavioral tests, only the forced swimming test scores showed significant improvement in the L‐DOPA‐treated group compared to that of MPTP‐induced PD model mice. In contrast, mice treated with 0.1 mg kg^−1^ NanoMassage demonstrated significant enhancements in all behavioral tests, highlighting the therapeutic potential of NanoMassage. Meanwhile, mice receiving 0.05 mg kg^−1^ NanoMassage exhibited improvements in the rotarod test and forced swimming tests, suggesting that the therapeutic efficacy of intranasal NanoMassage administration is dose dependent, consistent with observations following intra‐cerebroventricular injection. TH immunohistochemistry was conducted to examine the effects of intranasal administration of NanoMassage on dopaminergic neuronal function (Figure 7c). Our results show that mice administered with 0.1 mg kg^−1^ NanoMassage intranasally exhibited a significantly greater number of TH‐positive neurons in both the SN and the VTA compared to the MPTP group (Figure 7d). These findings indicate that intranasal administration of NanoMassage effectively mitigated MPTP‐induced dopaminergic neuronal loss.

To investigate the optimal treatment schedule for NanoMassage, MPTP‐induced PD model mice were administered by NanoMassage in three different schedules to mimic intervention at different stages of PD: i) early stage, an intranasal administration of 0.1 mg kg^−1^ NanoMassage on days 2 and 6; ii) middle stage, an intranasal administration of 0.1 mg kg^−1^ NanoMassage on day 4; and iii) late stage, an intranasal administration of 0.1 mg kg^−1^ NanoMassage on day 6. Motor functions were evaluated on day 8 using a series of behavioral tests, including the pole test, rotarod test, suspension test, and forced swimming test (Figure 7e). As expected, an intranasal administration of 0.1 mg kg^−1^ NanoMassage on days 2 and 6 resulted in optimal improvement in motor function, as evidenced by prolonged rotarod performance compared to mice administered NanoMassage on day 4 or 6 (Figure 7f). Mice receiving an intranasal administration of 0.1 mg kg^−1^ NanoMassage on day 6 exhibited significant improvement only in the forced swimming test. These results indicate that the therapeutic efficacy of NanoMassage can be optimized by adjusting treatment schedules. Not surprisingly, earlier intervention of NanoMassage would obtain better therapeutic benefit.

## 3 Discussion and Conclusion

In summary, our present study introduces a novel approach for PD treatment, by implementing the massage‐mimicking nanosheets within deep brain areas of lesion sites, providing valuable insights into the applications of advanced nanomaterials in disease therapy. To generate a suitable “NanoMassage,” we have screened biodegradable PEG‐BPNS with varied sizes and found that the lateral size of the PEG‐BPNS largely determined the interaction between them and neural cells. PEG‐BPNS with diameters of around 200 nm exhibited strong adherence to the surface of the neural cell membrane, as the “NanoMassage.” This effect facilitated the reorganization of actin filaments, consequently regulating the interactions between actin filaments, ER, and mitochondria, ultimately inhibiting mitochondrial fission and mitigating PD pathology through noninvasive administration. In contrast to existing methods that depend on altering ECM properties, utilizing specialized devices, or employing magnetic fields, our approach offers a readily implementable strategy for noninvasive delivery of mechanical stimulation to cells in deep brain regions for the treatment of neurological disorders, without disturbing healthy tissues.

For the treatment of diseases occurring in the brain, prior studies have focused on adjusting the structural/topographical complexity and surface chemistry of BPNS. For example, BPNS were modified with PEG to protect them from oxygen and water, significantly improving their stability in vivo.^[^ ^52^ ^]^ Additionally, under near‐infrared (NIR) irradiation, BPNS penetrate the BBB to protect cells from Cu^2+^ dyshomeostasis and oxidative‐stress‐induced cell apoptosis.^[^ ^28^ ^]^ Conjugating the brain‐targeting ligand lactoferrin and paeoniflorin onto the surface of BPNS protects neurons against 6‐hydroxydopamine‐induced dopaminergic neurotoxicity, further effectively treating PD in a targeted manner.^[^ ^53^ ^]^ However, these applications are mainly based on the electron transparency, optical/magnetic properties, or high drug loading of BPNS due to large surface area, but their other inherent bioactivities have rarely been investigated. Our study demonstrates that the inherent bioactivity of PEG‐BPNS is also size dependent. The size of PEG‐BPNS largely determined the interaction dynamics between PEG‐BPNS and cell membranes. PEG‐BPNS of various dimensions adhere to the PM of cells, with the 200 nm variant causing a significant increase in PM tension. Drawing insights from the composition and structure of PEG‐BPNS, we speculated that the distinct impacts of PEG‐BPNS on membrane tension regulation stem primarily from variations in the strength of hydrogen bond interactions, electrostatic attractions, and hydrophobic interactions with the phospholipid groups present in the PM of cells. The heightened accumulation of NanoMassage on the cell membrane, coupled with an extended duration of interaction in comparison to other sizes, fosters a robust and enduring interaction between NanoMassage and the cell membrane, leading to a notable escalation in membrane tension. Therefore, NanoMassage could regulate inter‐organelle contacts by producing mechanical stimuli. Notably, NanoMassage, which adhere directly to the cell membrane rather than being internalized, prevent potential cytotoxicity generated by elevated phosphate anions, acting similarly to the massage therapy.

Inter‐organelle contacts intricately regulate the morphology, quantity, and spatial distribution of organelles within cells. Disruptions in inter‐organelle contacts contribute to mitochondrial pathologies, encompassing aberrant ROS production, defective mitochondrial biogenesis, and disturbances in mitochondrial dynamics and quality control mechanisms. Despite ongoing efforts, the development and application of drugs targeting mitochondria remain in their infancy. Prominent agents include those aimed at targeting NAD^+^ metabolism, such as nicotinamide mononucleotide, as well as stabilizing the components of electron transport and ATP generation of mitochondria, such as coenzyme Q10 and Bendavia, along with mitochondria‐targeted antioxidants like mitoquinone and cyclosporin A, an inhibitor of the mitochondrial permeability transition pore.^[^ ^54^ ^]^ However, mitochondria do not exist in isolation within the cell but rather cooperate with other organelles to undergo a continuous cycle of fusion and fission, forming larger mitochondria that subsequently undergo fission to generate smaller entities. Mitochondrial fragmentation takes place at MERCs. DRP1 binds at these contact sites through actin filament polymerization, and the ER structurally contributes to mitochondrial fission by wrapping itself around the fission site, which is closely relevant to PD pathology.^[^ ^55^ ^]^ Therefore, there is an urgent need to develop drugs that can modulate inter‐organelle contacts, particularly the contacts between actin filaments, ER and mitochondria. In this study, we demonstrate a novel strategy for PD treatment by generating massage‐mimicking effect on the cell surface in deep brain regions to modulate inter‐organelle contacts.

In comparison to conventional strategies that predominantly target specific receptors for PD treatment, the concept of the “NanoMassage” as biomaterial at the nanoscale offers a unique approach. By mimicking the mechanical stimulation akin to massage therapy on the cell surface, NanoMassage trigger a timely increase of mechanical forces in the cellular environment. Consequently, this may facilitate the reorganization of actin filaments, thereby influencing the interactions among actin filaments, ER and mitochondria within the cell. Comparing to other nanosheets, such as graphene oxide and titanium dioxide, which show promise in biomedical applications,^[^ ^56^ ^]^ PEG‐BPNS have fewer active functional groups (e.g., carboxyl, hydroxyl, and epoxy groups).^[^ ^57^ ^]^ This potentially limits protein corona formation and allows PEG‐BPNS to precisely regulate inter‐organelle contacts without interference from proteins. Additionally, the mechanical properties of PEG‐BPNS make them sufficiently flexible to adapt to the dynamic cell membrane,^[^ ^58^ ^]^ facilitating effective actin reorganization, subsequently modulating MERCs and improving mitochondrial dynamics. Comparing to existing artificial tethers with persistently enhancement in MERCs in the context of cardiac muscle,^[^ ^59^ ^]^ NanoMassage provide dynamical mechanical stimulation that improves mitochondrial fission and fusion, as well as organelle interactions in neural cells for treating PD. This kind of dynamical MERCs’ enhancement allows for temporary modulation of organelle interactions based on specific cellular needs in a controlled manner without disturbing the basic cellular processes that support long‐term function, allowing cells to adapt to specific and transient stimuli. The balance between enhancing MERCs to improve cellular function and avoiding potential toxicity or dysfunction is delicate, and careful consideration is needed to ensure that chronic reprogramming does not inadvertently lead to further cellular damage or pathology. The dynamical MERCs’ enhancement provided by NanoMassage minimally reduces cellular plasticity, impairs adaptive responses to stress, and avoids long‐term organ‐level effects. Additionally, the “NanoMassage” approach allows for targeted delivery to deep brain regions without surgery, making it a potentially valuable approach for patients with PD, particularly those with difficulty accessing conventional treatments targeting deep brain regions.

Given the demonstrated effects of NanoMassage in modulating inter‐organelle contacts and mitochondrial dynamics, NanoMassage emerge as a promising therapeutic approach for addressing diseases associated with defects in MERCs, including familial PD,^[^ ^60^ ^]^ as well as conditions exacerbated by a high‐fat diet (HFD)^[^ ^61^ ^]^ and aging.^[^ ^62^ ^]^ Familial forms of PD, particularly those linked to mutations in *PINK1* and *PARKIN*, are characterized by impaired mitochondrial quality control, dysregulated MERCs, and disrupted mitochondrial dynamics.^[^ ^4^ ^]^ These defects contribute to mitochondrial dysfunction, increased oxidative stress, and, ultimately, neuronal degeneration.^[^ ^63^ ^]^ In addition to genetic factors, environmental and age‐related influences, such as HFD and aging, have been shown to exacerbate mitochondrial dysfunction and dopaminergic degeneration in PD.^[^ ^64^ ^]^ Research indicates that both HFD and aging dysregulate MERCs and mitochondrial dynamics, leading to oxidative stress, inflammation, and metabolic dysregulation, which further compromise mitochondrial function and neuronal survival.^[^ ^61^, ^65^ ^]^ These effects are particularly pronounced in the context of familial PD, where pre‐existing genetic mutations already impair mitochondrial homeostasis. Restoring the integrity of MERCs and mitochondrial dynamics represents a promising therapeutic strategy for these conditions. By enhancing the physical and functional interactions between mitochondria and the ER, NanoMassage have the potential to mitigate the detrimental effects of genetic mutations, environmental stressors, and aging, which provide a novel avenue for treating diseases associated with MERC dysfunction. In the realm of biomedical research, PEG‐BPNS have shown therapeutic efficacy in cancer and neurological diseases through various mechanisms, such as photothermal/photodynamic effects, chemotherapeutic anticancer effects, and binding of phosphorus to metal ions.^[^ ^28^, ^66^ ^]^ Diverging from previous research, our study not only offers a promising avenue for disease treatment but, crucially, introduces the concept of finely adjusting the size of nanosheets for the rational design. This adjustment enables precise control of mechanical force, providing a novel strategy for the regulation of organelle dynamics.

Mechanical properties at the cellular and tissue levels play a pivotal role in the pathogenesis of diseases.^[^ ^67^ ^]^ Biological mechanical cues, including hydrostatic pressure, tensile force or stretching force, fluid shear stress, extracellular matrix stiffness or tissue elasticity, and extracellular fluid viscosity, can trigger a cascade of molecular events in cells at lesion areas.^[^ ^68^ ^]^ Specifically, mechanical cues induce the change of tension within cells, activating downstream cascades such as reactive oxygen species generation, further affecting the proliferation, survival, and apoptosis of cells.^[^ ^35^ ^]^ In the process, mechanosensors within cells detect and convert these mechanical forces into physiological responses.^[^ ^69^ ^]^ Integrins, cadherin, Piezos, and G‐protein‐coupled receptors are known to convert mechanical signals into biochemical signals through force‐induced conformational changes.^[^ ^30b^ ^]^ Understanding the correlation between different mechanical stimuli produced by diverse nanomaterials and alterations in cell behavior presents a substantial challenge that requires further exploration. Investigating the conformational changes of these mechanosensors in response to various nanomaterials offers an efficient approach to analyzing the impact of nanomaterials on cell behavior and unveiling the fundamental principles governing changes in cell behavior triggered by mechanical stimuli. In this study, we have observed size‐dependent effects of PEG‐BPNS on PM tension; it is essential to understand potential size‐related variations in circulation, including the formation of a protein corona.^[^ ^70^ ^]^ Different dimensions of nanosheet may lead to the binding of diverse adhesive proteins, subsequently eliciting varied cellular responses by altering the total surface area that cells can perceive.^[^ ^71^ ^]^ Despite the challenges encountered, our research offers a unique strategy that insights into the potential therapeutic application of massage‐mimicking materials in brain disease management.

## 4 Experimental Section

### Materials

Black phosphorus nanosheet dispersion liquid was purchased from Nanjing MKNANO Tech and MOPHOS and stored in a dark vacuum glovebox. Amino‐group‐functionalized PEG (NH_2_─PEG2000─NH_2_) was purchased from Beijing JenKem Technology Co., Ltd. PBS (pH 7.4), 4‐hydroxyethyl piperazine ethanesulfonic acid (HEPES) buffer, Hanks balanced salt solution, 1 × tris‐buffered saline with tween‐20 (TBST); Radioimmunoprecipitation assay buffer (RIPA) lysis buffer and protease inhibitor cocktail were purchased from Beijing Solarbio Biotechnology Co., Ltd. Fetal bovine serum was purchased from Biological Industries (USA). Dulbecco's modified eagle medium, penicillin and streptomycin (PS) were purchased from Gibco (USA). Texas Red‐X Conjugated wheat germ agglutinin was purchased from Thermo (USA). Retinoic acid, TritonX‐100, bovine serum albumin (BSA), MPP^+^, and 3‐(4,5‐dimethyl‐2‐thiazolyl)‐2,5‐diphenyl‐tetrazolium bromide (MTT) were purchased from Sigma–Aldrich (USA). CCCP, oligomycin, Y27632, LIMKi3, and L‐DOPA were purchased from MedChemExpress (MCE, USA). Mitotracker Green was purchased from Invitrogen (USA). DCFH‐DA was purchased from Beijing Dingguo Changsheng Biotechnology Co., Ltd. Phalloidin (red and far red) and Mitotracker Red were purchased from YEASEN (China). Phosphate Sensor Assay Kit, Hoechst 33342, TUNEL apoptosis assay kit, and mitochondrial membrane potential assay kit with JC‐1 were purchased from Beyotime Biotechnology (China). YAP antibody (sc‐101199) and TOM20 antibody (sc‐136211) were purchased from Santa Cruz (USA). Tyrosine hydroxylase antibody (NB300‐109) was purchased from Novus Biologicals (USA). PSD95 antibody (ab18258) was purchased from abcam (USA). β‐ACTIN antibody (BE0021) was purchased from EASYBIO (China). Phospho‐DRP1 (Ser616) antibody (4494S) was purchased from Cell Signaling Technology (USA). OPA1 antibody (No. R382025), DRP1 antibody (No. 221099), and SPIRE1 antibody (No. 125232) were purchased from ZEN BIO (China). INF2 antibody (#20466‐1‐AP), IR3R3 antibody (#20729‐1‐AP), GRP75 antibody (#67563‐1‐Ig), and VDAC1 antibody (#66345‐1‐Ig) were purchased from ProteinTech (China). Goat anti‐mouse immunoglobulin G (IgG) (H&L)‐horseradish peroxidase (HRP)‐conjugated (BE0102) and goat anti‐rabbit IgG (H&L)‐HRP‐conjugated (BE0101) were purchased from EASYBIO (China). Tetramethyl rhodamine isothiocynate (TRITC) AffiniPure goat anti‐mouse IgG (H+L) (115‐025‐003), fluorescein (FITC) AffiniPure goat anti‐mouse IgG (H+L) (115‐095‐003), and Alexa Fluor 647 AffiniPure goat anti‐rabbit IgG (H+L) (111‐605‐003) were purchased from Jackson (USA). DAPI was purchased from Fanbo Biochemical Co. (USA).

### Nanomaterial Synthesis and Characterization

Black phosphorus nanosheet dispersion liquid was centrifuged at 1000 rpm to remove large particles for 15 min and the supernatant was centrifuged at 1500 × *g* for 10 min to collect 500 nm BPNS. The sediment of BPNS was resuspended into water and the supernatant was centrifuged at 6000 × *g* for 10 min to collect 200 nm BPNS. The sediment of BPNS was resuspended into water and the supernatant was centrifuged at 20 000 × *g* for 10 min to collect 100 nm BPNS. PEG (2 mg) was dispersed into BPNS solution (20 mL, 20 µg mL^−1^) for 30 min and sonicated. After stirring for 4 h, the mixture was centrifuged and washed two times at 20 000 × *g* for 20 min to remove excess PEG molecules and PEG‐BPNS were obtained. The morphology of the BPNS and PEG‐BPNS was obtained by Talos L120C G2 TEM (FEI, USA) operated at an accelerating voltage of 120 kV. The hydrodynamic diameter and zeta potential measurements were determined by dynamic light scattering (Malvern, UK). The FTIR spectra were performed by a Bruker TENSOR 37 FTIR analyzer (Bruker, Germany).

### Cell Culture

The human neuroblastoma cell line SH‐SY5Y was purchased from the American Type Culture Collection (ATCC). SH‐SY5Y cells were incubated in high‐glucose DMEM supplemented with 10% FBS and 1% PS in an incubator at 37 °C, 95% air and 5% CO_2_. SH‐SY5Y cells were treated with 10 µm RA for 72 h to induce the differentiation of cells. Treatment of differentiated SH‐SY5Y cells with 2.5 µm CCCP, 10 µm oligomycin, or 250 µm MPP^+^‐induced mitochondrial damage and showed PD phenotype.

### In Vitro Cytotoxicity Assay

SH‐SY5Y cells were plated in 96‐well plates at 4 × 10^4^ cells per well and incubated overnight. Then, gradient concentrations of BPNS and PEG‐BPNS were incubated with cells for 24 h. 100 µL of MTT solution (1 mg mL^−1^) was added into each well to replace the medium and incubated for 4 h. After that, MTT solution was removed, and then 150 µL of dimethyl sulfoxide (DMSO) solubilized solution was added into each well and shaken for 10 min. Absorbance was monitored at 570 nm with the Multiskan GO microplate reader. Cell viability standard without any treatment served as a blank control.

### Quartz Crystal Microbalance Assay

The SH‐SY5Y cell membrane was prepared according to the method reported previously.^[^ ^72^ ^]^ Briefly, cells were harvested and resuspended in HEPES buffer solution supplemented with 1% protease inhibitor cocktail. Cells were disrupted with IKAT18 basic ULTRA‐TURRAX (IKA) and consequent cell membrane fragments were purified by discontinuous sucrose density gradient ultracentrifugation. The interfacial interactions between cell membranes and nanomaterials were then characterized using a QCM (Q‐Sense). 100 µL of cell membrane debris (0.2 mg mL^−1^) was spin‐coated onto Au chips. PEG‐BPNS were pumped in and interacted with the cell membrane. After the curve reached a relatively stable level, the deionized water was pumped and rinsed the unbound nanomaterials.

### In Vitro Cellular Uptake Assays

SH‐SY5Y cells were seeded in a 24‐well plate and cultured overnight. The cells were then treated with RdB/PEG‐BPNS for 0, 4, 8, and 12 h. The medium was then removed, and then cells were washed with PBS for twice and fixed in 4% paraformaldehyde (PFA) at 37 °C for 10 min. Then, cells were labeled by Texas Red‐X conjugated wheat germ agglutinin for 10 min at room temperature and stained with DAPI, and subjected to confocal laser scanning microscopy (Zeiss LSM 800, Germany).

### Membrane Tension Analysis

To determine the plasma membrane tension of SH‐SY5Y cells, the cultural medium of cells was replaced by the staining solution (1 µm Flipper‐TR, Cytoskeleton, USA). The cells were placed in the incubator at 37 °C in a humidified atmosphere containing 5% CO_2_ for 15 min before imaging. FLIM imaging was performed using a Nikon Eclipse Ti A1R microscope equipped with a time‐correlated single‐photon counting module from PicoQuant. For FLIM analysis, the SymPhoTime 64 software was used to fit the data according to a 2‐exponential reconvolution model and calculate the lifetime of the Flipper‐TR probe.

### Cumulative Phosphorus Analysis

To assess the cumulative phosphorus content in SH‐SY5Y cells resulting from the degradation of BPNS and PEG‐BPNS, the cells were treated with BPNS or PEG‐BPNS for varying durations (0, 4, 8, 12, and 24 h). Subsequently, the culture medium was removed, and the cells were washed thrice with PBS. The cells were then harvested, suspended in PBS, and disrupted utilizing a homogenizer. The resultant cellular lysate was centrifuged to obtain the cell supernatant. This supernatant was combined with a phosphate sensor according to the method of Phosphate Sensor Assay Kit (#S0192S, Beyotime, China) to quantify the phosphorus concentration in the cells.

### Immunofluorescence

RA was used to differentiate SH‐SY5Y cells into neuron‐like cells for 72 h. Then, cells were treated with MPP^+^ for 24 h. Cells were incubated with NanoMassage for 8 h after MPP^+^ stimulation for 16 h in the NanoMassage group. For mitochondria and actin cytoskeleton labeling, neuron‐like cells were incubated with Mitotracker green (#C1048, Beyotime Biotechnology, China) and Phalloidin (#40762ES75, YEASEN, China) for 30 min at 37 °C and stained with DAPI and subjected to confocal laser scanning microscopy (Zeiss LSM 800, Germany). For the staining of YAP, neuron‐like cells were washed two times with cold PBS, fixed in 4% paraformaldehyde at 37 °C for 10 min, blocked with goat serum at room temperature for 30 min and stained with anti‐YAP antibody (sc‐101199, Santa Cruz, USA) at 4 °C overnight. Cells were then washed three times with cold PBS and incubated with FITC goat anti‐mouse IgG (H+L) at room temperature for 1 h. Cells were then washed three times with cold PBS and stained with DAPI and subjected to confocal laser scanning microscopy (Zeiss LSM 800, Germany). For the inhibition of Rho/ROCK pathway, cells were treated with Y27632 (10 µm, HY‐10071, MCE, USA), LIMKi3 (20 µm, HY‐18305, MCE, USA) diluted in complete media for 24 h and then fixed and stained as described afterward.

### Mitochondria and Autophagosome Labeling and Analysis

Neuron‐like cells were transfected with RFP‐LC3 plasmid to label autophagosome. After transfection for 24 h, cells were incubated with Mitotracker green (#C1048, Beyotime Biotechnology, China) for 30 min at 37 °C. Then, cells were imaged using Zeiss Super‐Resolution Imaging System SIM and SMLM. The co‐localization index Mander's coefficients were calculated. Images were also reconstructed by Imaris (Bitplane) followed the manufacturer instructions.

### Electron Microscopy

Cells were harvested and fixed overnight at 4 °C using 2.5% glutaraldehyde in PBS. The cells were then washed twice with PBS and once with ddH_2_O. Subsequently, the cells were postfixed in 1% O_s_O_4_ and 1.5% K_3_Fe(CN)_6_ for 90 min at room temperature. The cells were then washed with ddH_2_O and incubated in chilled 2% aqueous uranyl acetate for 1 h at room temperature. Following a final wash with ddH_2_O, the cells were dehydrated through a graded ethanol series and embedded in epoxy EMBED‐812 resin (14120, Electron Microscopy Sciences). Images were acquired using a Hitachi H‐7800 electron microscope operating at 100 kV, with RADIUS 2.2 software at room temperature. The electron microscope was equipped with an AMT CCD camera (MoradaG3, EMSIS).

### Mitochondria and DRP1 Staining and Analysis

RA was used to differentiate SH‐SY5Y cells into neuron‐like cells for 72 h. Then, cells were treated with MPP^+^ for 24 h. Cells were incubated with NanoMassage for 8 h after MPP^+^ stimulation for 16 h in the NanoMassage group. For mitochondria and DRP1 protein labeling, neuron‐like cells were incubated with Mitotracker red (#40740ES50, Yeasen, China) for 30 min at 37 °C and washed two times with cold PBS, fixed in 4% paraformaldehyde at 37 °C for 10 min, blocked with goat serum at room temperature for 30 min and stained with anti‐DRP1 antibody (sc‐101270, Santa Cruz, USA) at 4 °C overnight. Cells were then washed three times with cold PBS and incubated with FITC goat anti‐mouse IgG (H+L) at room temperature for 1 h. Cells were then washed three times with cold PBS and stained with DAPI and subjected to confocal laser scanning microscopy (Zeiss LSM 800, Germany).

### Mitochondria and Endoplasmic Reticulum Staining and Analysis

SH‐SY5Y cells were transfected with ER–mCherry plasmid to label the structural network of the endoplasmic reticulum. RA was used to differentiate SH‐SY5Y cells into neuron‐like cells for 72 h. Then, cells were treated with MPP^+^ for 24 h. Cells were incubated with NanoMassage for 8 h after MPP^+^ stimulation for 16 h in the NanoMassage group. The mitochondrial structural network was detected by 50 nM Mitotracker green (#C1048, Beyotime Biotechnology, China) in a culture medium at 37 °C for 45 min. Then, cells were imaged using a Zeiss Super‐Resolution Imaging System SIM and SMLM. Acquired images for mitochondria morphology were analyzed. The co‐localization index Mander's coefficients were calculated. MERCs were also reconstructed and analyzed via the surface–surface contact site area tool by Imaris (Bitplane) followed the manufacturer instructions.

### Mitochondrial Membrane Potential (△*ψ*_m_)

RA was used to differentiate SH‐SY5Y cells into neuron‐like cells for 72 h. The △*ψ*_m_ of neuron‐like cells was determined by assay kit with JC‐1 (#C2006, Beyotime Biotechnology, China). Neuron‐like cells with or without NanoMassage preincubation for 8 h were treated with CCCP (#HY‐100941, MCE, USA) for 20 min or oligomycin (#HY‐N6782, MCE, USA) for 1 h. Cells were harvested and washed with cold PBS twice. JC‐1 working stock was incubated with cells at 37 °C for 20 min in the dark. Cells were then washed with JC‐1 buffer solution for two times. Next, cells were incubated in Hoechst 33342 for counterstaining of cell nuclei and observed with confocal laser scanning microscopy (Zeiss LSM 800, Germany).

### Intracellular ROS Determination

The intracellular ROS was quantified as the previous report.^[^ ^73^ ^]^ Fluorescence imaging was performed to detect intracellular ROS levels by DCFH‐DA (#S0033S, Beyotime Biotechnology, China), which produces strong fluorescence upon encountering intracellular ROS. The intensity of DCF fluorescence was positively correlated with the amount of intracellular ROS. In detail, the cells preincubated with or without NanoMassage for 8 h were treated with CCCP for 20 min or oligomycin for 1 h. Subsequent to removing the medium, the cells were conditioned with 10 µm DCFH‐DA for 1 h. Next, cells were incubated in Hoechst 33342 for counterstaining of cell nuclei and analyzed by confocal laser scanning microscopy (Zeiss LSM 800, Germany) at the end.

### TUNEL Staining

Cells were fixed with 4% paraformaldehyde for 30 min at room temperature and washed twice with PBS. The cells were then incubated in the TUNEL solution containing FITC–dUTP for 60 min at 37 °C according to the manufacturer's protocol (#C1088, Beyotime Biotechnology, China). Next, cell nuclei were labeled by DAPI. Images were captured and analyzed using confocal laser scanning microscopy (Zeiss LSM 800, Germany).

### Animals’ Experiments

C57BL/6J male mice, 10–12 weeks of age, were obtained from Charles River Laboratories (Beijing, China). All animal studies were conducted under the guidelines set by the Tianjin Committee of Use and Care of Laboratory Animals, and the overall project protocols were approved by the Animal Ethics Committee of Nankai University (approval ID: 2021‐SYDWLL‐000318).

### MPTP‐Induced Mouse PD Model and Treatment

Briefly, a mouse model of PD was constituted by intraperitoneal injection of MPTP (5 mg mL^−1^) at a dose of 25 mg kg^−1^ for 7 days. Mice were anesthetized by an intraperitoneal injection of 3.5% chloral hydrate. The mice were then immobilized on a brain stereotaxic instrument (Stoelting, 51500D, USA). The scalp was incised and a hole was drilled at a specific location on the skull (1.0 mm laterally from the anterior fontanelle and 0.2 mm posteriorly). A 5 µL Hamilton syringe with a needle was then inserted through this hole into the lateral ventricle 2.5 mm below the level of bregma. Different concentrations of NanoMassage or saline (2 µL) were injected into the left ventricle, respectively. For intranasal administration of NanoMassage, awaken mice were fixated, and small drops of 10 µL NanoMassage probes were applied with a pipettor in front of the nasal cavity and inhaled by the mice. After 4 h of injection, mice (*n* = 3 independent mice per group) were sacrificed and dissected, and the IVIS fluorescence imaging system was utilized by placing the main organs on the equipped platform (Ex = 546 nm and Em = 568 nm). For the administration of L‐DOPA (HY‐N0304, MCE, USA), 20 mg kg^−1^ L‐DOPA was intragastrically administrered into mice through a gavage needle. The needle was inserted into the mouth of mice, guiding it along the roof of the oral cavity and down the esophagus. Proper alignment was ensured to avoid tracheal insertion. The needle was advanced gently until the predetermined depth was reached, corresponding to the stomach. Slowly the syringe plunger was depressed to deliver the substance into the stomach. Rapid administration was avoided to prevent reflux or aspiration.

### Rotarod Test

All mouse behavioral instruments were purchased from the Zhishuduobao (Beijing, China) biotechnology company. The assessment was carried out on a mouse accelerated rotating rod device (rotating rod diameter: 3 cm). The rotating rod device was accelerated at a steady speed of 1–25 rpm for 300 s and the time taken for each test was recorded. The test ended when the mouse fell off the rotating rod or when the time reached 300 s. A rest of 180 s was allowed between each test.

### Forced Swimming Test

Ahead of time, 1.5 L of water was poured into a 2 L beaker and placed in a water bath and the temperature of the water in the beaker was controlled between 25–26 °C using a thermometer. The scoring criteria were as follows: 3.0 points for mice that swam continuously for 1 min; 2.5 points for mice that swam most of the time and only occasionally floated; 2.0 points for mice that floated for more than 50% of the entire test time; 1.5 points for mice that swam occasionally; 1.0 point for mice that occasionally swam with their hind limbs and floated on one side; and 0.0 points for no movement of the four limbs.

### Suspension Test

Mice were hung by their front paws on a horizontally placed piece of twine 30 cm above the ground. The mouse scores 3.0 points for grasping the twine with both hind paws, 2.0 points for grasping the twine with one hind paw, 1.0 point for failing to grasp the twine with both hind paws and 0.0 points for the mouse falling off.

### Pole Test

A straight rod with a diameter of 0.8 cm and a height of about 60 cm was used for the experiment. The top of the pole had a small wooden ball covered with gauze to prevent the mice from slipping. The mouse was placed head up on top of the pole and the sum of two times was recorded: the time from the start of the mouse's movement until fully head down and the time to climb to the bottom of the pole.

### Immunoblotting

Total protein lysate from neuron‐like cells and mouse SN were extracted with RIPA lysis buffer, sonicated on ice, and centrifuged at 13 400 × *g* at 4 °C. TH (1:1000, #NB300‐109, Novus Biologicals), PSD95 (1:1000, ab18258, abcam), DRP1 pS616 (1:1000, #4494S, Cell Signaling Technology), DRP1 (1:1000, No. 221099, ZEN BIO), SPIRE1 (1:1000, No. 125232, ZEN BIO), INF2 (1:1500, #20466‐1‐AP, ProteinTech), OPA1 (1:1000, No. R382025, ZEN BIO), IR3R3 (1:500, #20729‐1‐AP, ProteinTech), GRP75 (1:1000, #67563‐1‐Ig, ProteinTech), VDAC1(1:5000, #66345‐1‐Ig, ProteinTech), or β‐ACTIN (1:5000, #BE0021, EASYBIO), and goat anti‐mouse IgG (H&L)‐HRP Conjugated (1:5000, #BE0102, EASYBIO) or goat anti‐rabbit IgG (H&L)‐HRP conjugated (1:5000, #BE0101, EASYBIO) antibodies were used. Data quantification was performed by ImageJ software (version 1.53).

### Preparation of RdB/PEG‐BPNS and RdB/NanoMassage

To prepare RdB/PEG‐BPNS and RdB/NanoMassage, 10 µL of RdB solution (4 mg mL^−1^) was added to 200 µg of PEG‐BPNS or NanoMassage solution and reacted in the dark for 12 h. Then the mixture solution was centrifuged three times at 20 000 × *g* for 30 min to remove the unreacted RdB.

### Immunohistochemistry

Brain sections were collected from healthy mice or MPTP‐induced PD model mice 1 day after administration of RdB/NanoMassage on the 4^th^ day of MPTP administration. Brain sections were collected from healthy mice or MPTP‐induced PD model mice 4 days after intra‐cerebroventricular injection or intranasal administration of NanoMassage on the 4^th^ day of MPTP administration. After behavioral tests, mice with intra‐cerebroventricular administration of saline or NanoMassage were randomly divided into two subgroups: three mice for immunohistochemistry and six mice for western blot. In immunohistochemistry, brain slices were placed in 50 °C oven for 30 min. Then, slices were washed with TBST (Solarbio) three times for 10 min and blocked in 0.4% TritonX‐100 (Sigma), BSA (Solarbio) (blocking buffer) for 1 h, followed by overnight incubation with the primary antibody including anti‐TH (1:200, NB300‐109, Novus Biologicals), anti‐TOM20 (1:200, sc‐136211, Santa Cruz) at 4 °C. Then, recycling the primary antibody, rinsing with 1 × TBST three times for 10 min, and then sections were incubated for 1 h at room temperature in a mixture of FITC AffiniPure goat anti‐mouse IgG (H+L) (1:200, 115‐095‐003, Jackson), Phalloidin (#40762ES75, YEASEN), or Alexa Fluor 647 AffiniPure goat anti‐rabbit IgG (H+L) (1:200, 111‐605‐003, Jackson). Rinsing sections with 1 × TBST three times for 10 min, single‐stained slides were dropped with glycerol containing DAPI (Fanbo Biochemical Co.), double‐stained slides were dropped with 50% glycerol, and mount. Images were captured using confocal laser scanning microscopy (Zeiss LSM 800, Germany).

### Data Analysis

Statistical comparisons were made by unpaired *t*‐test, one‐way ANOVA or two‐way ANOVA (for multiple comparisons), and all data were acquired from at least three independent samples. *p* < 0.05 was considered statistically significant. All statistical calculations were displayed using GraphPad Prism (version 9.0.0).

## Conflict of Interest

The authors declare no conflict of interest.

## Supporting information



Supporting Information

## Data Availability

The data that support the findings of this study are available from the corresponding author upon reasonable request.
