# Supplementary material for: Massage‐Mimicking Nanosheets Mechanically Reorganize Inter‐organelle Contacts to Restore Mitochondrial Functions in Parkinson's Disease
Source: Adv Sci (Weinh). 2025 Apr 13;12(20):2413376. doi: 10.1002/advs.202413376 (PMC12120710; doi:10.1002/advs.202413376)
Supplement: Supplementary file 1 — Supporting Information [file ADVS-12-2413376-s001.docx]

Supporting Information for

**Massage-mimicking Nanosheets Mechanically Re-organize Inter-organelle Contacts to Restore Mitochondrial Functions in Parkinson's Disease**

Tianqi Li, Liwen Huang, Chenxiao Guo, Jing Ren, Xi Chen, Yachu Ke, Zengyu Xun, Wenzhuo Hu, Yilin Qi, Heping Wang, Xing-Jie Liang, Xue Xue*

T. Li, L. Huang, C. Guo, J. Ren, X. Chen, Y. Ke, Z. Xun, W. Hu, Y. Qi, H. Wang, Prof X. Xue

State Key Laboratory of Medicinal Chemical Biology, College of Pharmacy, Nankai University, Tianjin, PR China

Email: xuexue@nankai.edu.cn

X.-J. Liang

Laboratory of Controllable Nanopharmaceuticals, Chinese Academy of Sciences (CAS) Center for Excellence in Nanoscience and CAS Key Laboratory for Biomedical Effects of Nanomaterials and Nanosafety, National Center for Nanoscience and Technology, Beijing, PR China

University of Chinese Academy of Sciences, Beijing, PR China

L. Huang

Current address: Department of Chemistry, Shanghai Key Laboratory of Molecular Catalysis and Innovative Materials, State Key Laboratory of Molecular Engineering of Polymers and iChem, Fudan University, Shanghai, PR China

H. Wang

Current address: State Key Laboratory of Advanced Medical Materials and Devices, Tianjin Key Laboratory of Radiation Medicine and Molecular Nuclear Medicine, Key Laboratory of Radiopharmacokinetics for Innovative Drugs, Tianjin Institutes of Health Science, Institute of Radiation Medicine, Chinese Academy of Medical Sciences & Peking Union Medical College, Tianjin, PR China

**
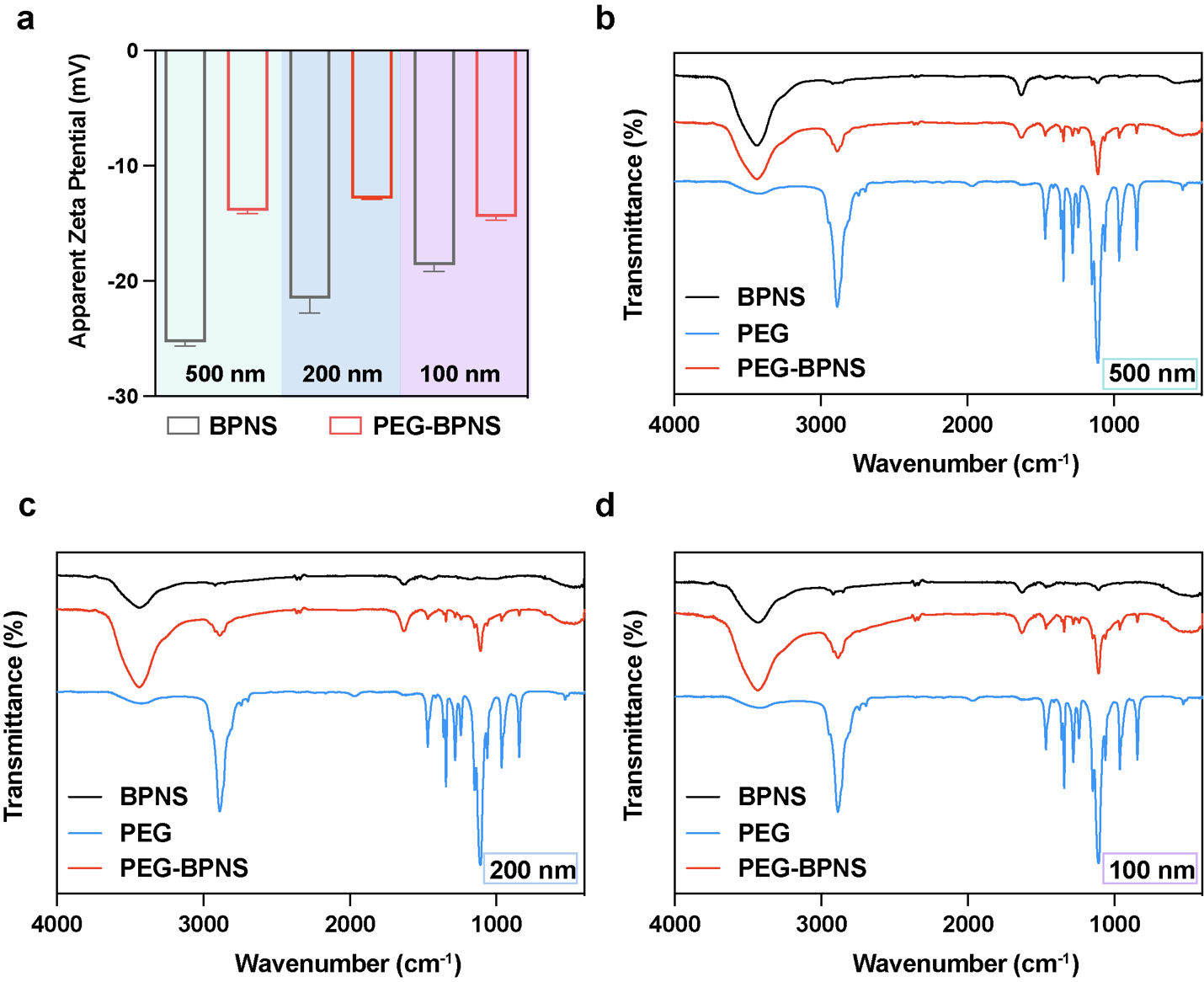
**

Figure S1. Characterization of black phosphorus nanosheet (BPNS) and PEGylated black phosphorus nanosheet (PEG-BPNS). (a) Zeta potential analysis of BPNS and PEG-BPNS with gradient sizes. Data are expressed as means ± SEM (n = 3). (b-d) Fourier transform infrared spectroscopy (FTIR) spectra of BPNS, PEG and PEG-BPNS with gradient sizes.


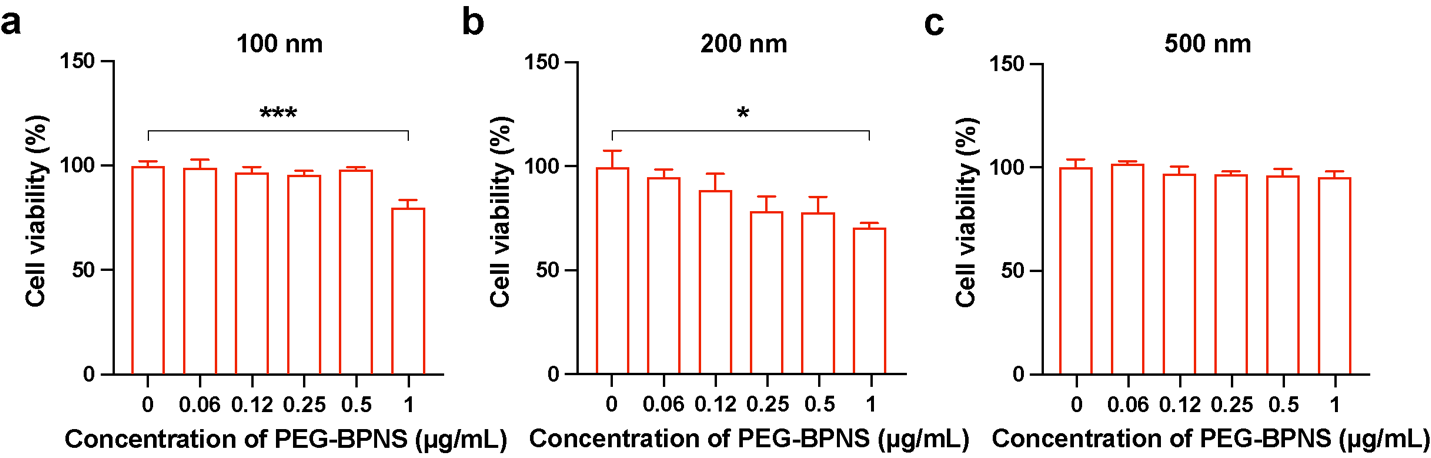


Figure S2. Cell viability test of PEG-BPNS measured by 3-(4,5-Dimethylthiazol-2-yl)-2,5-diphenyltetrazolium bromide (MTT). Neural cells were incubated with gradient concentrations of PEG-BPNS with gradient sizes for 24 h to determine the maximum use concentration (n = 3 biological replicates). Data are presented as means ± SEM. Statistical significance was determined by one-way ANOVA with a Dunnett's multiple comparisons test. **P* < 0.05 and ****P* < 0.001.

**
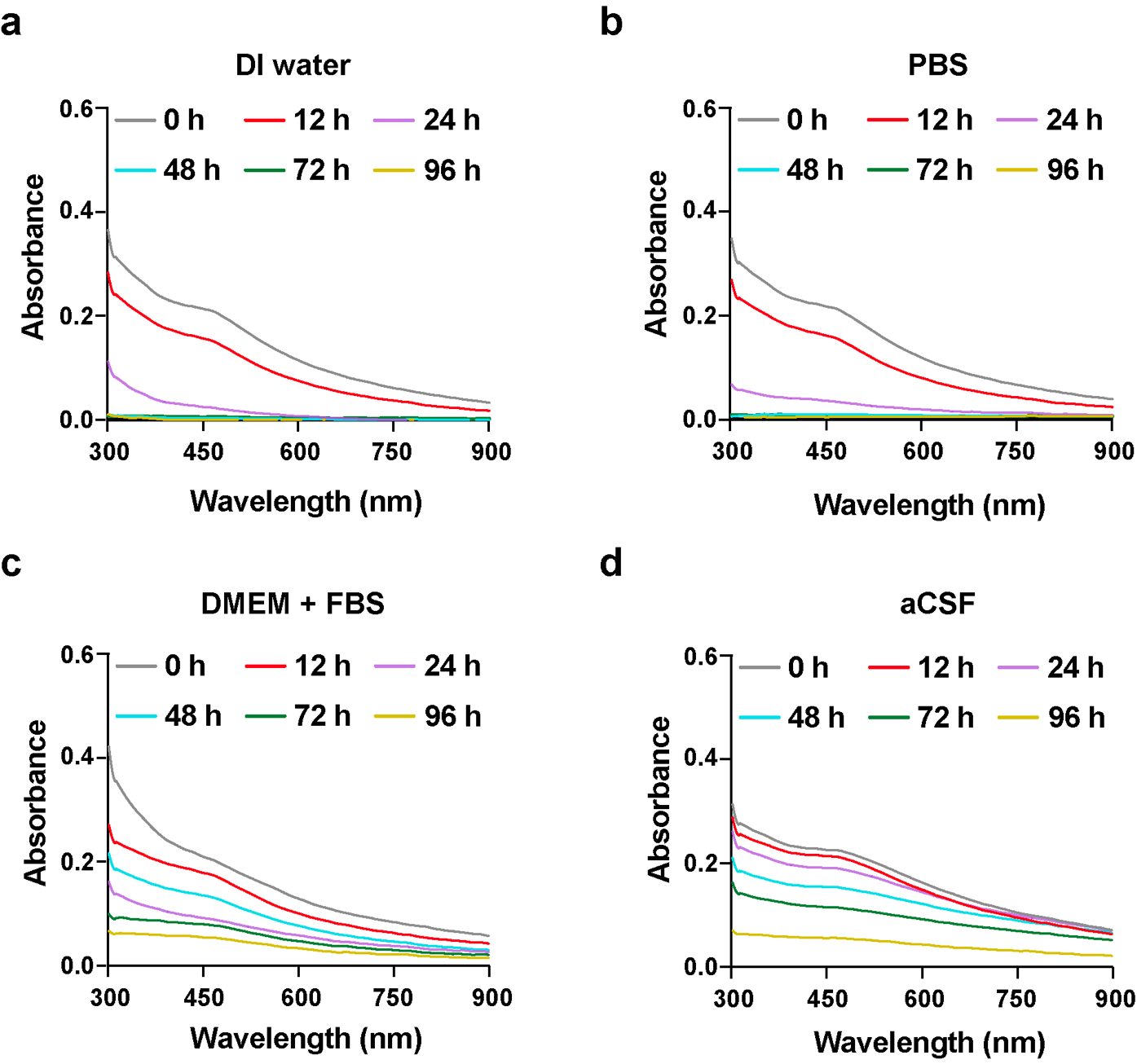
**

**Figure S3.** Evaluation of the stability of 200 nm PEG-BPNS in physiological conditions. Absorption spectra of 200 nm PEG-BPNS dispersed in air-exposed DI water (a), PBS (b), DMEM containing 10% FBS (c) and artificial cerebrospinal fluid (aCSF) (d) at 37ºC for 0, 12, 24, 48, 72, and 96 h. An obvious decrease in absorbance was observed of 200 nm PEG-BPNS in different solutions for gradient times.


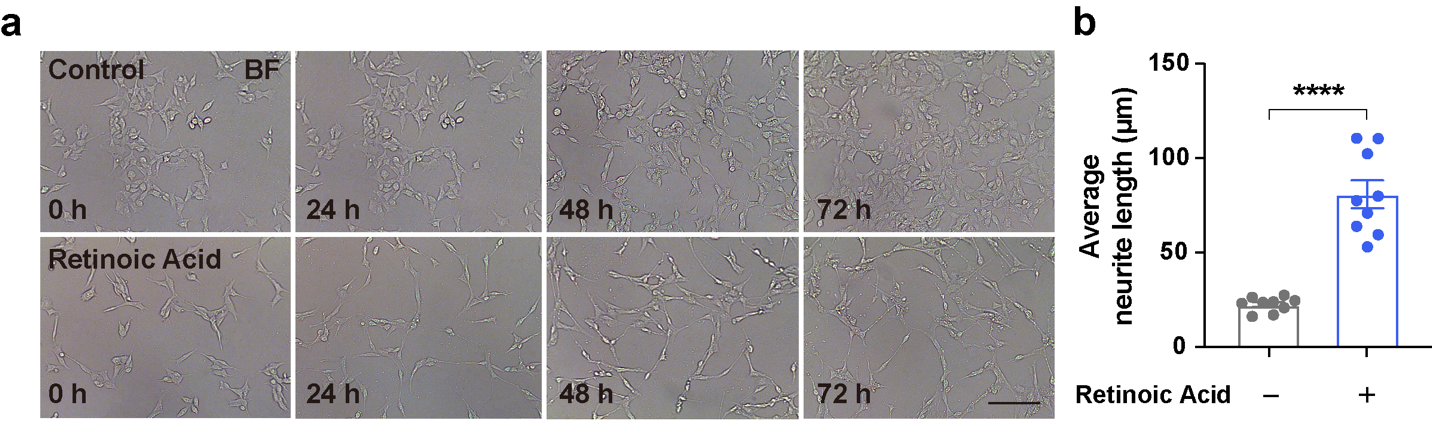


Figure S4. SH-SY5Y cells differentiate into neuron-like cells following 72 h of incubation with retinoic acid (RA). (a) Bright-field microscopic images of SH-SY5Y cells with or without RA stimulation were recorded continuously for 72 h. Scale bar, 100 μm. (b) Analysis of average neurite length of cells treated with or without RA for 72 h (n = 9 fields from 3 biological replicates). Data are presented as means ± SEM. Statistical significance was determined by an unpaired t-test. *****P* < 0.0001.

**
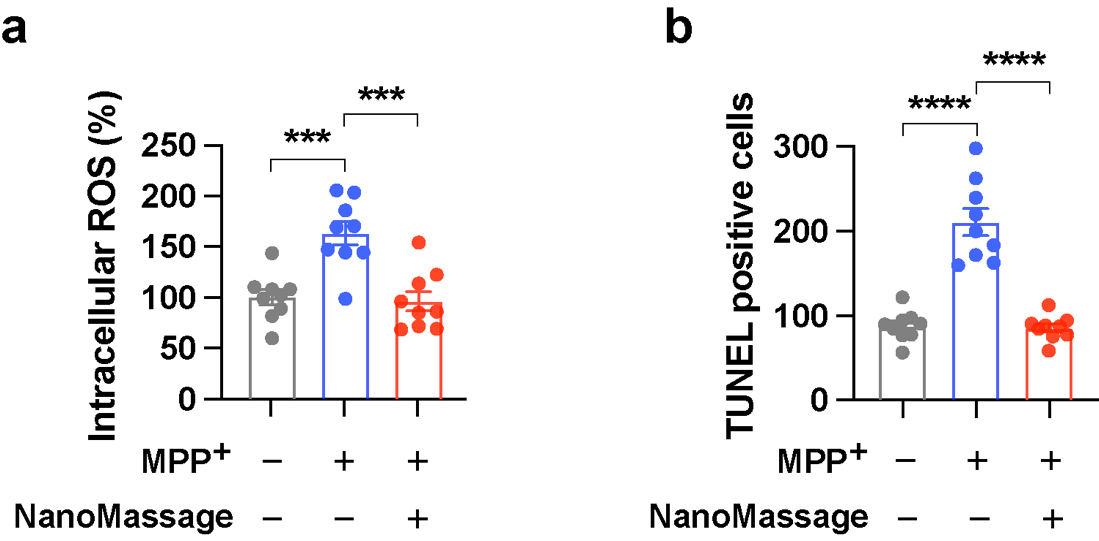
**

**Figure S5.** NanoMassage reduce ROS generation and inhibit cellular apoptosis in neuron-like cells. (a) Quantification of intracellular ROS levels in neuron-like cells with indicated treatments (n = 9 fields from 3 biological replicates). (b) Quantification of TUNEL-positive neuron-like cells with indicated treatments (n = 9 fields from 3 biological replicates). Data are shown as means ± SEM. Statistical significance was determined by one-way ANOVA with a Dunnett's multiple comparisons test. ****P* < 0.001 and *****P* < 0.0001**.**


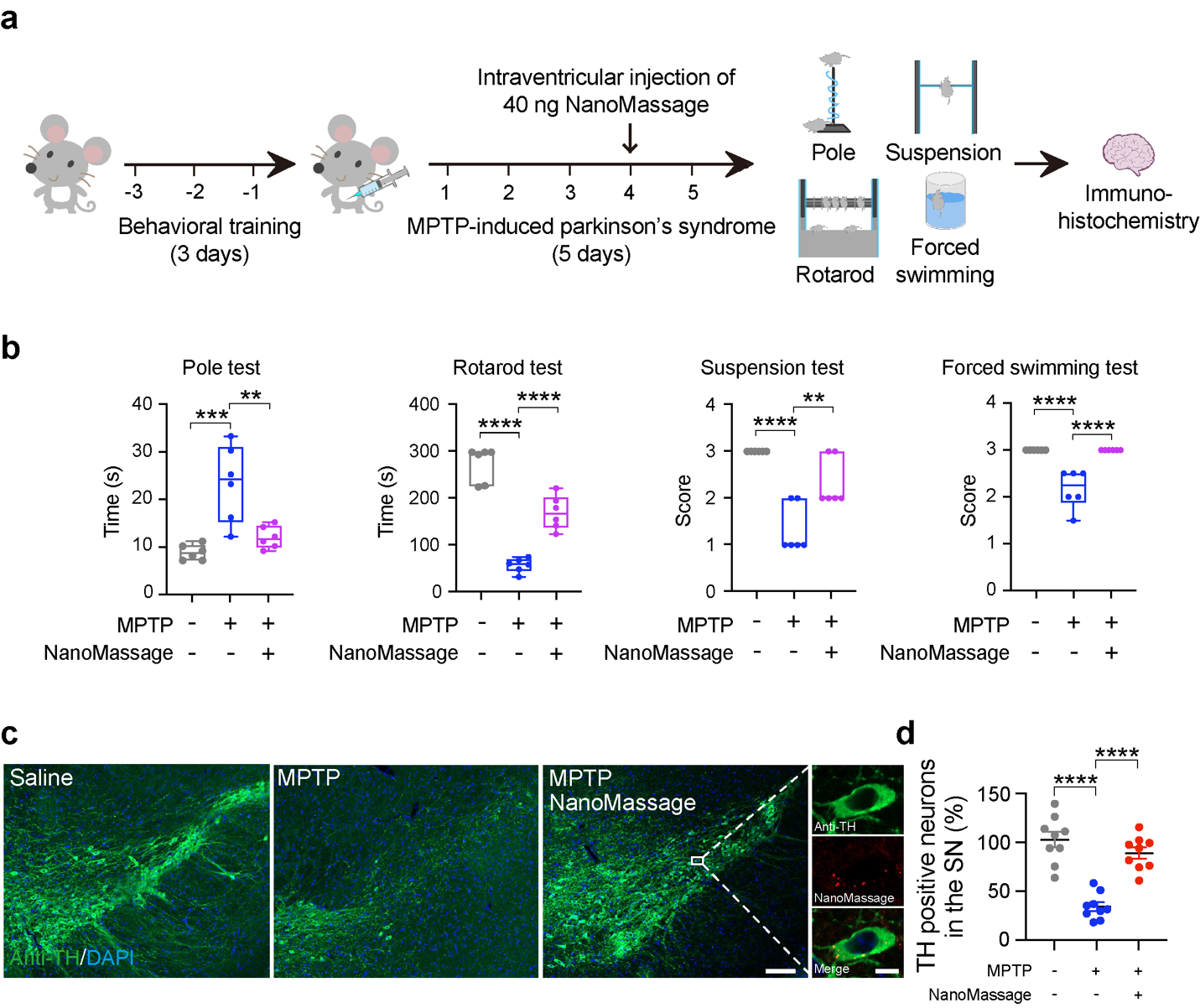


**Figure S6.** NanoMassage exhibit protective effect on dopaminergic neurons in mice following 24 h administration. (a) Treatment schedule for the MPTP‐induced mouse model of PD with or without RdB/NanoMassage. Mice were behaviorally trained for 3 days prior to producing experimental parkinsonism by MPTP. MPTP was administered intraperitoneally for a continuous period of 7 days (25 mg/kg, once a day) to induce the disease model. Mice were given an intracerebroventricular injection of 40 ng of RdB/NanoMassage or equal volume saline on the 4^th^ day of MPTP administration. On the 5^th^ day, mice were sacrificed and dissected after four different behavioral tests as the schematic shows. (b) Behavior analysis of pole test, rotarod test, suspension test and forced swimming test for mice administered 40 ng of RdB/NanoMassage or saline (n = 6 independent mice/group). (c) Representative fluorescence images of dopaminergic neurons (anti-TH, green) in the SN of mice administered 40 ng of RdB/NanoMassage or saline. Cell nuclei were stained with DAPI (blue). Scale bar, 200 μm. (d) Graph shows the percentages of TH-positive neurons in the SN of mice. n = 9 independent fields from 3 mice/group. Data are shown as means ± SEM. Statistical significance was determined by one-way ANOVA with a Dunnett's multiple comparisons test. ***P* < 0.01, ****P* < 0.001 and *****P* < 0.0001.


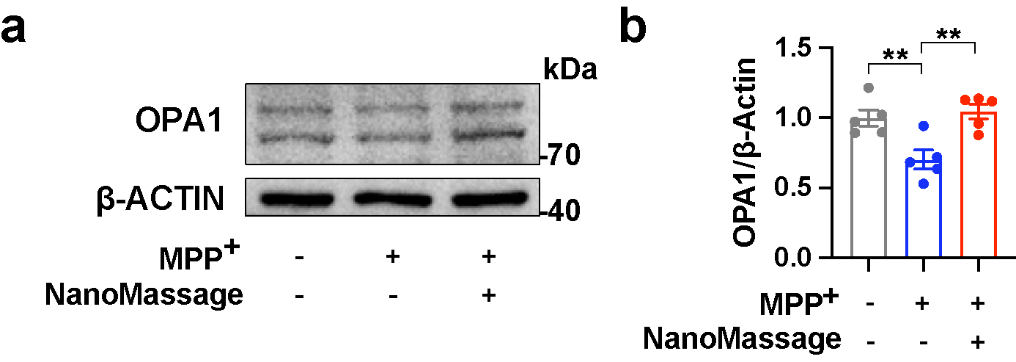


Figure S7. NanoMassage increase OPA1 level in neuron-like cells with MPP^+^ stimulation. Western blot analysis (a) and quantification (b) of OPA1 expression in neuron-like cells with different treatments. n = 5 biological replicates. The values were normalized to the control group. Data are shown as means ± SEM. Statistical significance was determined by one-way ANOVA with a Dunnett's multiple comparisons test. ***P* < 0.01.


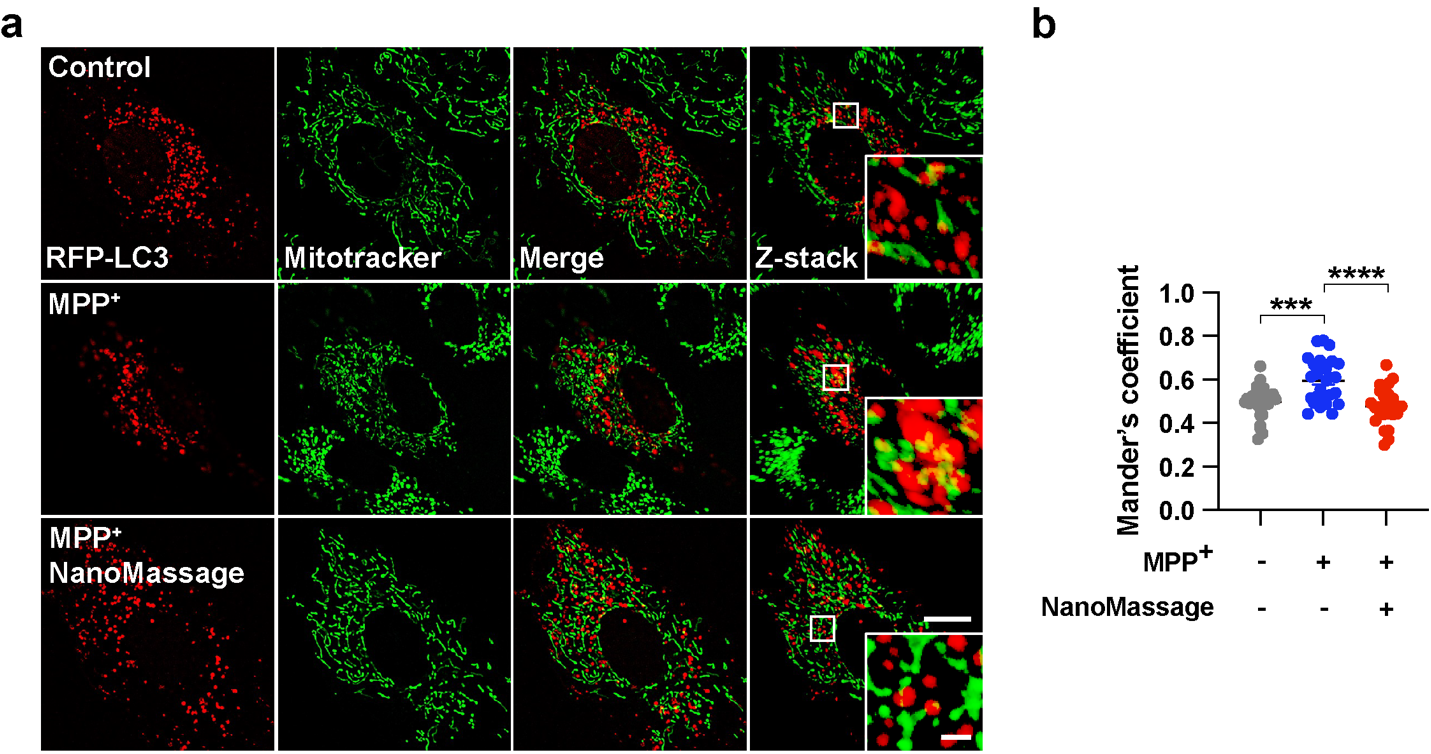


**Figure S8.** NanoMassage mitigate the excessive mitophagy induced by MPP^+^ in neuron-like cells. (a) Representative structure illumination microscopy (SIM) images of colocalization of autophagosome (RFP-LC3, red) and mitochondria (Mitotacker, green) in neuron-like cells. Z-stack images were acquired and deconvolution images are presented as maximum intensity z-projection. Scale bars: 10 μm (original images) and 2.5 μm (zoomed-in images). (b) The fraction of RFP-LC3 puncta that overlaps with Mitotracker labeled mitochondria in cells was quantified by Mander’s colocalization coefficient in 27 cells from 3 independent experiments. Data are presented as means ± SEM. Statistical significance was determined by one-way ANOVA with a Dunnett's multiple comparisons test. ****P* < 0.001 and *****P* < 0.0001.


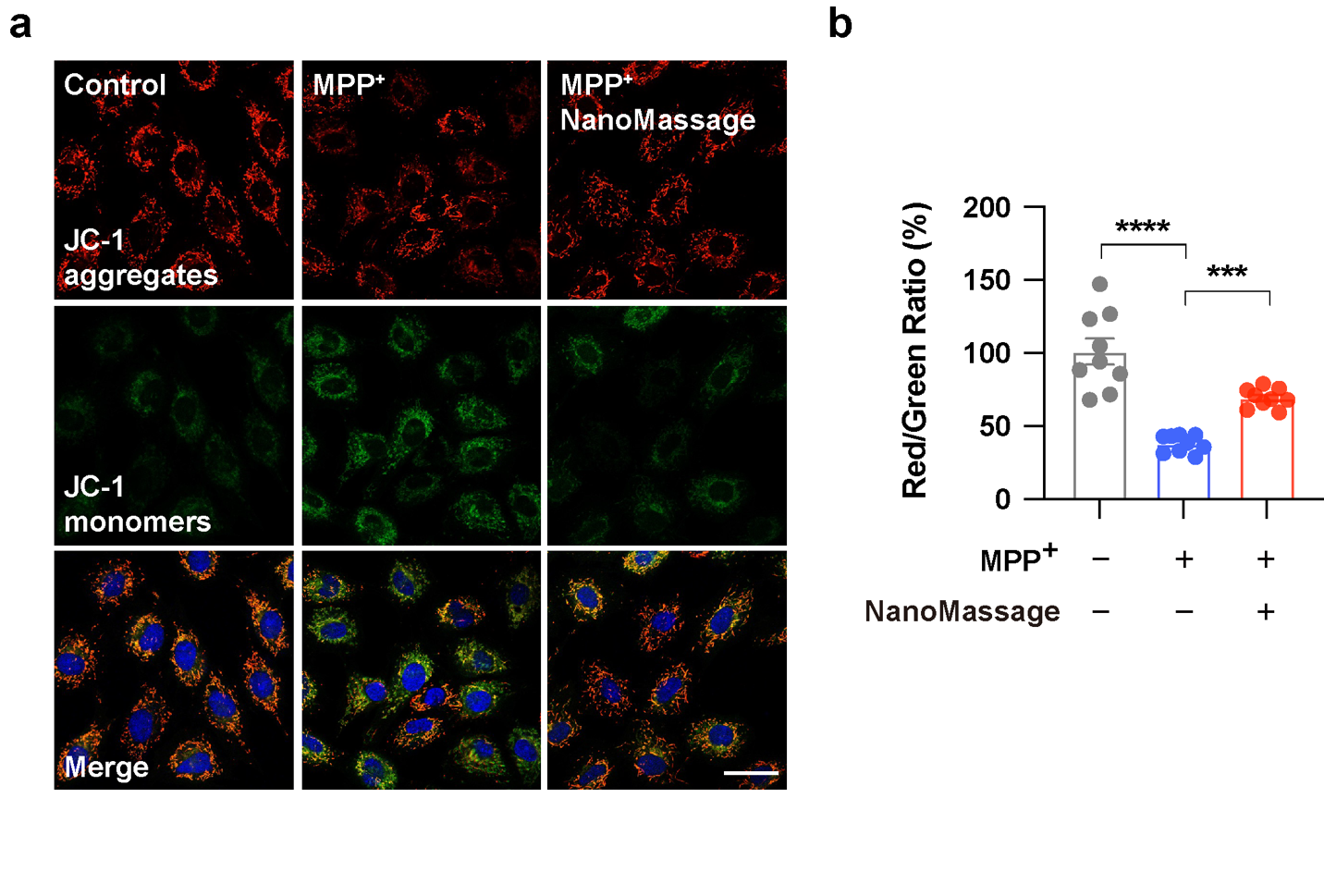


Figure S9. NanoMassage maintain the mitochondrial membrane potential of neuron-like cells with MPP^+^ stimulation. (a) Assessment of mitochondrial membrane potential (△ψ_m_) by JC-1 staining. Mitochondria were stained with JC-1, and cell nuclei were stained with Hoechst 33342 (blue). Scale bar, 20 μm. (b) △ψ_m_ determined by JC-1 assay (n = 9 fields from 3 biological replicates). The ratio of the red fluorescence intensity to the green fluorescence intensity was analyzed using Image J and is shown in the bar chart. Data are presented as means ± SEM. Statistical significance was determined by one-way ANOVA with a Dunnett's multiple comparisons test. ****P* < 0.001 and *****P* < 0.0001.

**
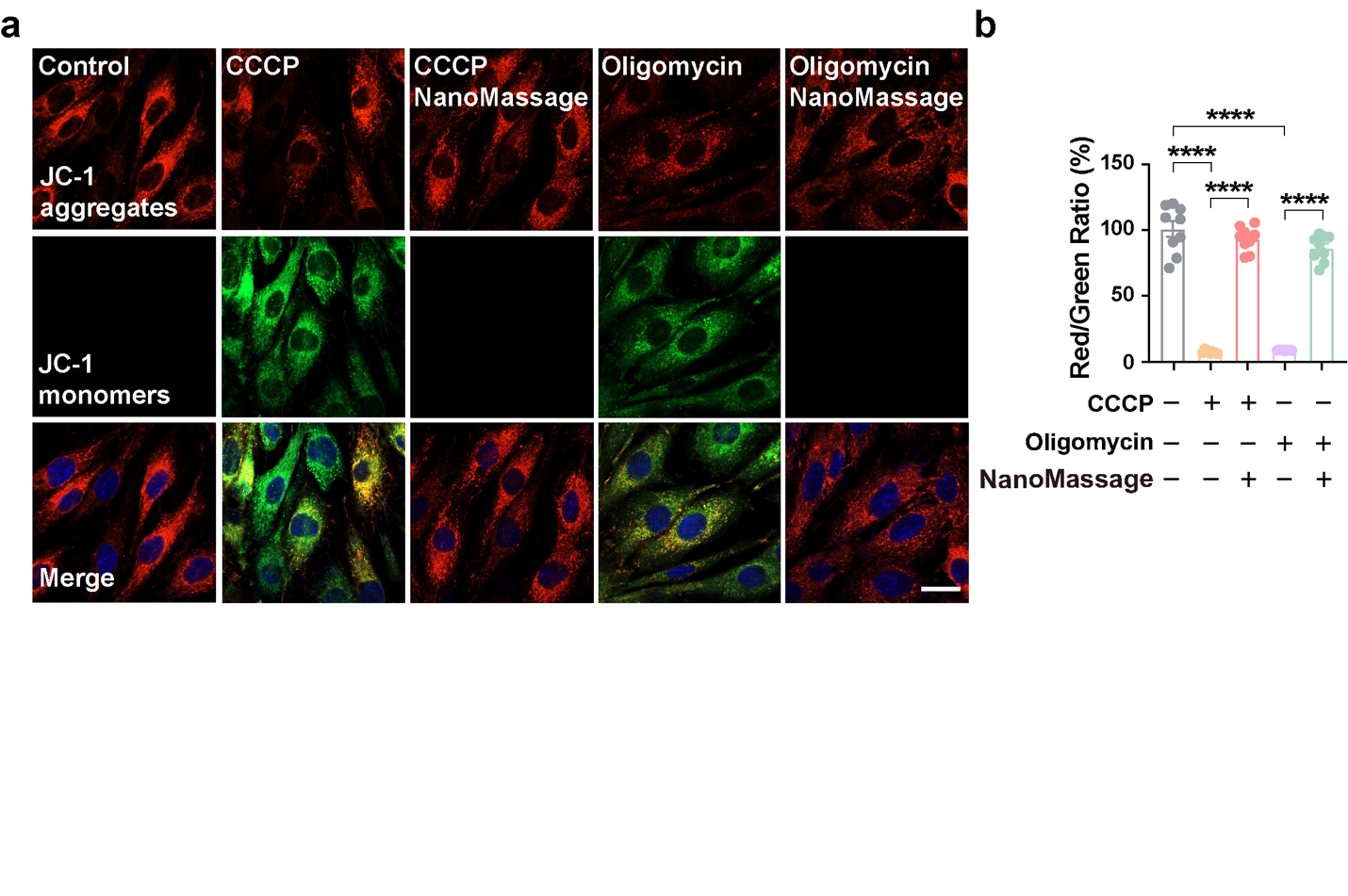
**

Figure S10. NanoMassage maintain the mitochondrial membrane potential of neuron-like cells with CCCP or oligomycin stimulation. (a) Assessment of △ψ_m_ by JC-1 staining. Mitochondria were stained with JC-1, and cell nuclei were stained with Hoechst 33342 (blue). Scale bar, 20 μm. (b) △ψ_m_ determined by JC-1 assay (n = 9 fields from 3 biological replicates). The ratio of the red fluorescence intensity to the green fluorescence intensity was analyzed using Image J and is shown in the bar chart. Data are presented as means ± SEM. Statistical significance was determined by one-way ANOVA with a Šídák's multiple comparisons test. *****P* < 0.0001.

**
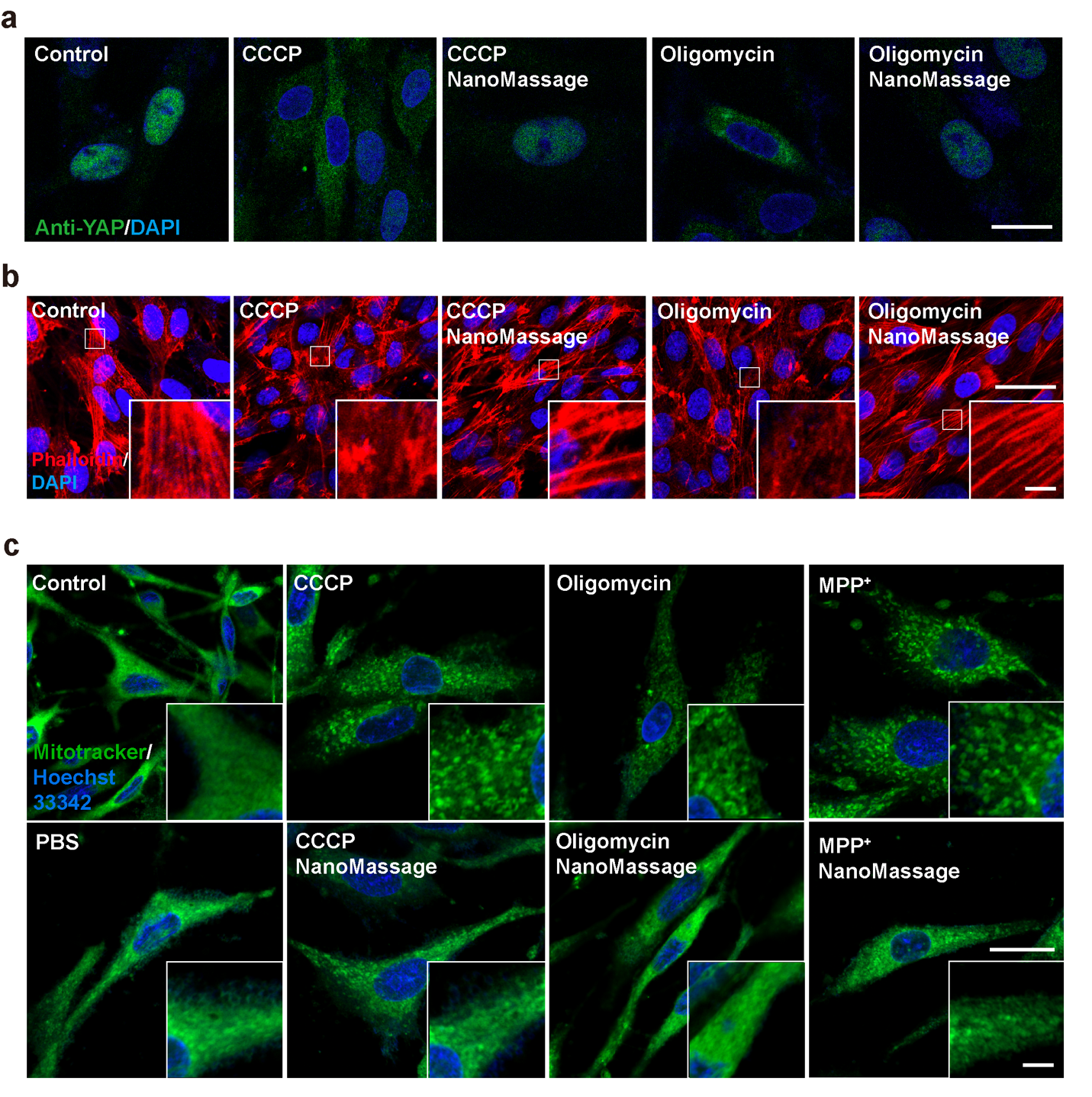
**

Figure S11. NanoMassage induce the translocation of YAP and reorganization of actin filaments in neuron-like cells with CCCP or oligomycin treatment and protect mitochondrial network in neuron-like cells with CCCP, oligomycin or MPP^+^ treatment. (a) Confocal immunofluorescence images of YAP (anti-YAP, green) and nuclei (DAPI, blue) showed the subcellular localization of YAP in neuron-like cells. Scale bar, 25 μm. (b) Confocal immunofluorescence images of the actin cytoskeleton (Phalloidin, red) and nuclei (DAPI, blue) showed the remodeling of cytoskeleton in neuron-like cells with NanoMassage treatment. Neuron-like cells were pretreated with NanoMassage for 8 h, and then incubated with CCCP for 10 min or Oligomycin for 1 h. Scale bars: 25 μm (original images); 5 μm (zoomed-in images). (c) Representative confocal images of mitochondria in neuron-like cells with indicated treatments. Mitochondria were labeled with Mitotracker (green), and cell nuclei were stained with Hoechst 33342 (blue). Scale bars: 25 μm (original images); 5 μm (zoomed-in images).

**
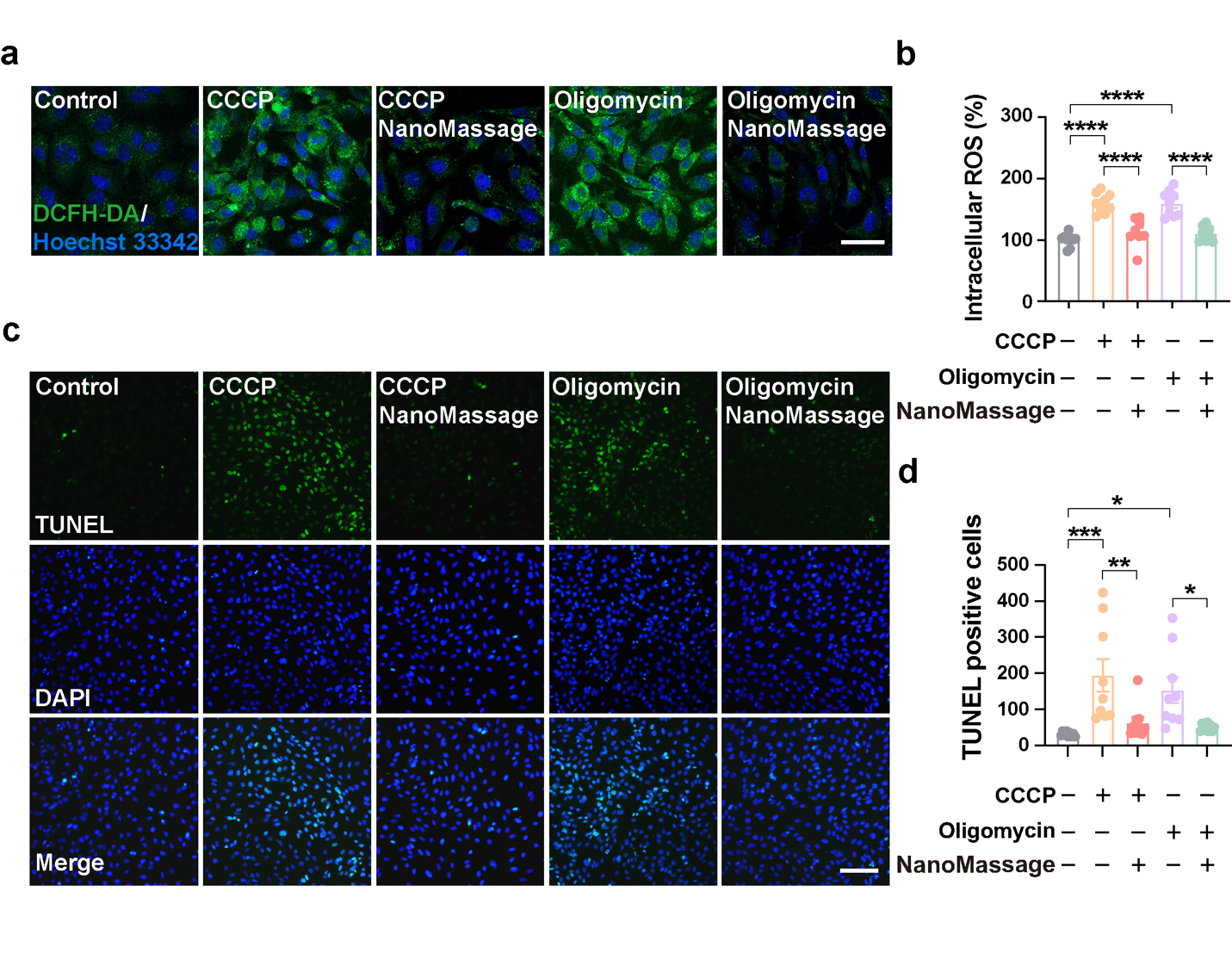
**

**Figure S12.** NanoMassage ameliorate ROS generation and cell apoptosis induced by CCCP or oligomycin in neuron-like cells. (a, b) Representative fluorescence images (a) and the quantification (b) of cellular ROS levels (stained by DCFH-DA probe, green) in neuron-like cells with indicated treatment (n = 9 fields from 3 biological replicates). Cell nuclei were stained with Hoechst 33342 (blue). Scale bar, 40 μm. (c) TUNEL staining of neuron-like cells with indicated treatments. Cell nuclei were stained with DAPI (blue). Scale bar, 50 μm. (d) Quantification of TUNEL-positive cells in different groups (n = 9 fields from 3 biological replicates). Data are presented as means ± SEM. Statistical significance was determined by one-way ANOVA with a Šídák's multiple comparisons test. **P* < 0.05, ***P* < 0.01, ****P* < 0.001 and *****P* < 0.0001.

**
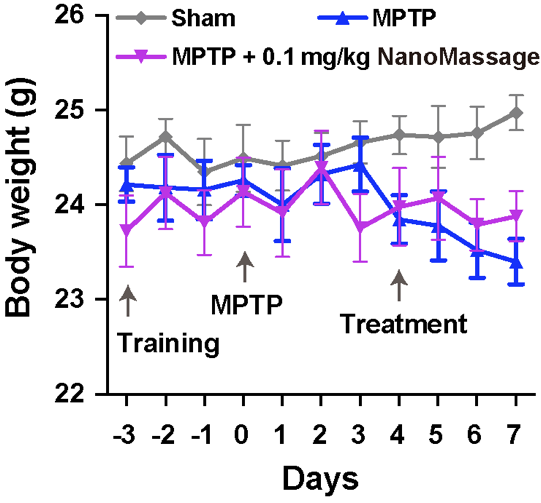
**

Figure S13. Body weights of mice with different administrations. n = 9 independent mice/group. Data are presented as means ± SEM.
